# Massively parallel genetic perturbation suggests the energetic structure of an amyloid-β transition state

**DOI:** 10.1126/sciadv.adv1422

**Published:** 2025-06-11

**Authors:** Anna Arutyunyan, Mireia Seuma, Andre J. Faure, Benedetta Bolognesi, Ben Lehner

**Affiliations:** ^1^ Wellcome Sanger Institute, Cambridge, UK.; ^2^ Institute for Bioengineering of Catalonia (IBEC), The Barcelona Institute of Science and Technology (BIST), Baldiri Reixac 10-12, 08028 Barcelona, Spain.; ^3^ Centre for Genomic Regulation (CRG), The Barcelona Institute for Science and Technology (BIST), Barcelona, Spain.; ^4^ Universitat Pompeu Fabra (UPF), Barcelona, Spain.; ^5^ Institució Catalana de Recerca i Estudis Avançats (ICREA), Barcelona, Spain.

## Abstract

Amyloid aggregates are pathological hallmarks of many human diseases, but how soluble proteins nucleate to form amyloids is poorly understood. Here, we use combinatorial mutagenesis, a kinetic selection assay, and machine learning to massively perturb the energetics of the nucleation reaction of amyloid-β (Aβ42), the protein that aggregates in Alzheimer’s disease. In total, we measure the nucleation rates of >140,000 variants of Aβ42 to accurately quantify the changes in free energy of activation of the reaction for all possible amino acid substitutions in a protein and, in addition, to quantify >600 energetic interactions between mutations. Strong energetic couplings suggest that the Aβ42 nucleation reaction transition state is structured in a short C-terminal region, providing a structural model for the reaction that may initiate Alzheimer’s disease. Using this approach it should be possible to reveal the energetic structures of additional amyloid transition states and, in combination with additional selection assays, protein transition states more generally.

## Teaser

The aggregation rates of >140,000 mutants reveal the energetic structure of an amyloid-β nucleation reaction transition state.

## INTRODUCTION

Amyloid fibrils are supramolecular fibrous protein assemblies in which β strands are stacked along the long axis of each fibril in an ordered “cross-β” structure (*1*). Specific amyloid assemblies define many human neurodegenerative diseases, including Alzheimer’s disease, Parkinson’s disease, and frontotemporal dementia. Fibrils of amyloid-β (Aβ42) are, for example, a pathological hallmark of Alzheimer’s disease and variants in Aβ42 cause familial Alzheimer’s disease (*2*, *3*). In total, at least 50 human disorders are associated with the formation of amyloid fibrils of more than 30 different proteins (*4*). Beyond human pathology, amyloids are present in all kingdoms of life and have functions in a wide variety of organisms, including humans (*5*).

Mature amyloid fibrils are stable structures and normally irreversible states. For many proteins, they are likely to be the thermodynamically most favored state at high protein concentration (*6*). The conversion of soluble proteins to amyloid fibrils occurs through nucleation-and-growth processes, with nucleation being the rate-limiting step (*7*). Most proteins do not form amyloids under physiological conditions because of kinetic control: The high free energy barrier of the nucleation reaction means fibrils never form on human timescales (*8*, *9*). Understanding why a small subset of proteins aggregate in human diseases therefore requires understanding nucleation reactions. Preventing nucleation would stop both the formation of mature fibrils and the production of potentially toxic fibril intermediates and oligomeric species produced during fibril assembly (*10*). Nucleation reactions are therefore the key processes to understand and prevent to stop amyloid formation and amyloid-associated toxicity in human diseases.

Thanks primarily to developments in cryo–electron microscopy (cryo-EM), the atomic structures of many amyloid fibrils have now been determined (*11*), including fibrils extracted from human brains (*12*). These structures have revealed that proteins can adopt different filament structures (fibril polymorphs) with, at least in some cases, different amyloid folds associated with different clinical conditions (*11*). Time-resolved cryo-EM has also been used to characterize the in vitro assembly of amyloids, revealing a diversity of folds that appear and disappear as fibrillation proceeds (*13*, *14*). Protein folding intermediates implicated in the initiation of amyloid formation have also been partially characterized by nuclear magnetic resonance, revealing native-like structures (*15*, *16*).

**Fig. 1.**
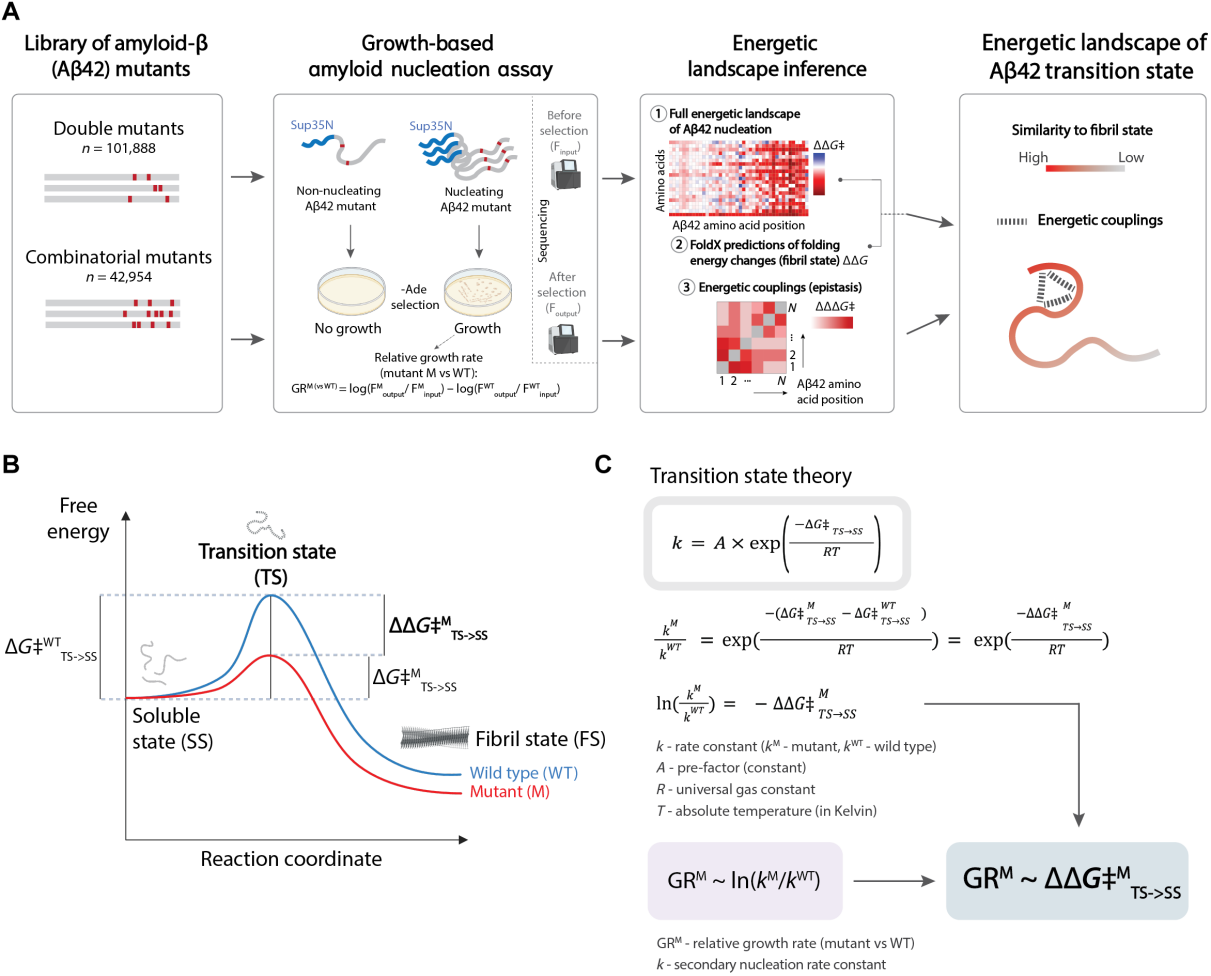
Quantifying changes in amyloid nucleation reaction activation energies at scale. (**A**) Schematic overview of the study: Mutant libraries are selected through an assay where cell growth depends on amyloid nucleation and is quantified by sequencing. The resulting relative growth rates are used to fit a mechanistic model to infer free energy of activation terms and energetic couplings, which can be used to map the energetic landscape of the Aβ42 transition state. (**B**) Free energy landscape of the amyloid nucleation reaction. (**C**) (Top) Transition state theory (*19*) equation relating the rate of a reaction with its free energy of activation and (bottom) derivation of the linear relationship between measured relative growth rates and change in free energy of activation; *k*, rate constant (*k*_*M*_ for mutant and *k*_*WT*_ for WT); A , the pre-factor; *∆*G*‡*, free energy of activation; *R*, universal gas constant; *T*, absolute temperature (kelvin); TS, transition state; FS, fibril state; SS, soluble state; *∆∆*G*‡*^*M*^, change in free energy of activation for mutant M (versus WT); *GR*^*M*^, relative growth rate (for mutant M versus WT).

The rate of a nucleation reaction depends on the difference in energy [the free energy of activation (**G*‡*)] between the highest energy state along the reaction coordinate (the transition state) and the initial soluble state (Fig. 1A) (*17*, *18*). Because of their high energy and transient nature, transition states are notoriously difficult to characterize. In contrast to folding intermediates (*13*–*16*), transition states have therefore not been structurally characterized for any amyloid nucleation reaction (*19*).

One disruptive approach for probing transition states, pioneered by Fersht and colleagues, is to use mutations (*20*–*22*). Mutations that stabilize a transition state (or increase the energy of the initial soluble state) will lower **G*‡* and accelerate a reaction, whereas mutations that destabilize the transition state will slow it. Kinetic measurements therefore allow the importance of individual residues and potential structural contacts in a transition state to be probed. Moreover, comparing changes in kinetics to changes in stability can provide information on the degree of native structure around a mutated residue in a transition state (*19*, *22*).

Using this approach, the transition states of enzymes and protein folding pathways have been probed using a small number of individual mutations (*23*–*25*), providing important structural insights (*20*, *21*). More recently, 10 mutations were used to probe the transition state of an amyloid elongation reaction, revealing that monomers that successfully bind to the fibril ends have fibril-like contacts (*26*). Another study combined molecular dynamic simulations with kinetic experiments to suggest a hairpin trimer structure for the transition state of Tau amyloids (*27*). However, in all cases, because of the difficulty of making kinetic measurements, only a very small number of carefully chosen mutations have been characterized.

More generally, the genotype-phenotype landscapes of protein aggregation reactions are largely unexplored. To address this shortcoming, we have developed methods that allow the nucleation kinetics of thousands of sequences to be quantified in parallel using pooled mutation-selection-sequencing experiments (*28*–*31*). This has opened up the possibility of exploring the genetic architecture of amyloid formation—the set of rules that govern how mutations combine to determine the rate of fibril assembly. It could be that amyloid genotype-phenotype landscapes are complex, requiring many parameters to accurately predict changes in nucleation when mutations are combined. Alternatively, nucleation genetics might be simple. We consider a genotype-phenotype landscape simple if it can be described using few parameters (*32*) (providing a large data compression) and parameters that are interpretable (providing understanding).

Here, we directly tackle the challenge of exploring amyloid genotype-phenotype landscapes, using Aβ42 as a model system. In total, we quantify the nucleation rates of >140,000 combinatorial mutants of Aβ42, including >100,000 double mutants and >40,000 higher-order combinations of mutations. Fitting energy models to this dataset using neural networks reveals a simple genetic architecture, with nucleation rates accurately predicted by additive changes in the free energy of activation and a contribution from rare pairwise energetic couplings. These simple energy models represent large compressions of the full genotype space—102- and 1581-fold for the models with and without energetic couplings, respectively. The strongest energetic couplings between mutations are, moreover, not random but strongly enriched between a subset of residues that form structural contacts in mature Aβ42 fibril structures. A simple interpretation of the energy landscape is that these contacts are already formed in the transition state of the nucleation reaction, predicting a structural model for the Aβ42 nucleation transition state. Using this approach, it should be possible to massively perturb the energy landscapes of additional nucleation reactions and, in combination with additional selection assays, the energetics of protein transition states more generally.

## RESULTS

### Quantifying the nucleation of >100,000 variants of Aβ42

The rate of an amyloid nucleation reaction depends exponentially on the free energy of activation [*∆*G*‡* (free energy difference between the transition and ground states)] of the reaction, as described by the transition state theory: $k=A\times \text{exp}\left(-\frac{\Delta G‡}{RT}\right)$ , where k is the nucleation rate constant, R is the universal gas constant, T is the absolute temperature, and A is the pre-factor, which, in the simplest form, can be estimated according to Kramers theory (*33*) as $A=\left(\frac{C}{\sigma +\eta }\right)$ , where C is a constant, η is the solvent viscosity, and σ is the “internal friction” of the protein (Fig. 1, B and C) (*19*, *34*). To quantify changes in k for all mutations in Aβ42, we used a kinetic selection assay in which the rate of Aβ42 nucleation controls the growth rate of yeast cells (Fig. 1A and fig. S1). Comparison with in vitro nucleation rate constants from different studies shows that relative growth rates are proportional to the logarithm of nucleation rate constants (*28*, *29*). However, the dynamic range of the assay is bounded by the maximum and minimum quantifiable relative growth rates, limiting measurement precision.

To expand the measurement range of the selection assay, we used double mutant libraries. Testing mutations in fast nucleating variants of Aβ42 provides an expanded dynamic range for quantifying decreased nucleation, whereas testing mutations in slow nucleating Aβ42 variants expands the dynamic range for quantifying increased nucleation. Using a large number of double mutants reduces the influence of specific interactions between mutations (*35*, *36*).

**Fig. 2.**
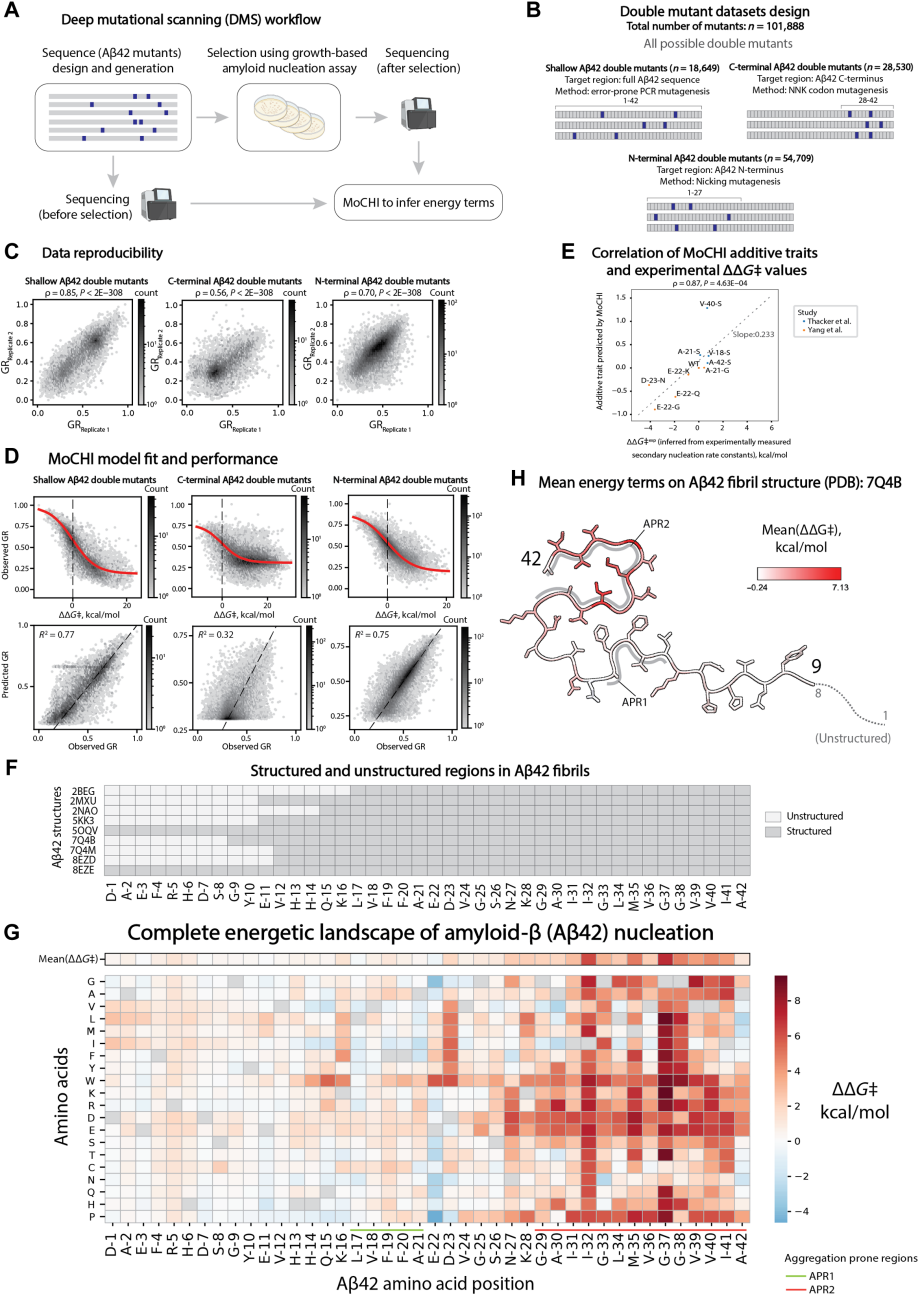
Complete free energy of activation landscape for Aβ42 single mutants. (**A**) Schematic overview of the deep mutational scanning (DMS) approach. (**B**) Aβ42 double mutant libraries’ design. (**C**) Inter-replicate correlations of relative growth rates (GR) for shallow, C-terminal and N-terminal Aβ42 double mutant libraries (from left to right); Spearman’s ρ (correlation) coefficients and associated *P* values are reported. (**D**) MoCHI model fit [(top) red trend line represents the sigmoid function fit; black vertical dashed line indicate 0 in the *x* axis] and correlation between observed and predicted relative growth rates (bottom) for shallow, C-terminal, and N-terminal Aβ42 double mutants datasets. (**E**) Scatterplot of additive trait values predicted by the MoCHI (*36*) model trained on double mutant datasets (*y* axis) and experimentally derived *∆∆*G*‡* values (*x* axis; derived from secondary nucleation rate constants) used to calibrate MoCHI-inferred terms to kcal/mol units (see Methods for details). Gray dashed line represents the linear regression fit for the data. (**F**) Overview of structured and unstructured regions in Aβ42 fibrils. (**G**) Complete energetic landscape of Aβ42 nucleation: heatmap of inferred free energy of activation terms (*∆∆*G*‡*) for all possible substitutions in Aβ42. Mean ∆∆*G*‡ values for each position are displayed in the top row of the heatmap (outlined in black). APR1 and APR2 are highlighted in light green and orange, respectively. (**H**) Cross section of the 7Q4B PDB structure of Aβ42 fibrils with residues colored by mean *∆∆*G*‡* per position. APR1 and APR2 are highlighted in gray.

In total, we quantified the relative growth rates of 101,888 double mutants of Aβ42 using three separate libraries and triplicate selection experiments (Fig. 2, A to C; fig. S2B; table S2; and Methods). Across these double mutants, the effect of each amino acid substitution is quantified in a median of 215 Aβ42 single mutants, providing measurements of changes in nucleation upon mutation in Aβ42 sequences with many different rates of nucleation.

### Changes in free energy of activation for all substitutions in Aβ42

We used MoCHI, a flexible toolkit for fitting models to deep mutational scanning data (*35*, *36*) to infer the change in free energy of activation (*∆∆*G*‡*), for all possible amino acid substitutions. The model assumes additivity of free energy changes across the entire double mutant dataset, with a sigmoidal function accounting for the upper and lower bounds of the relative growth rate measurements (Fig. 2D and fig. S2C). The resulting inferred free energies correlate well with free energies obtained using secondary nucleation rates from in vitro measurements (*37*, *38*) on unfused Aβ42 variants and which were used for linear scaling of the dataset (Fig. 2E, fig. S2A, and Methods). Adding the inferred free energy changes for each mutation provides good prediction of the double mutant nucleation rates (Pearson’s *R*^2^ = 0.72, evaluated by 10-fold cross-validation, total of 60,510 variants) (Fig. 2D and table S3). The very good performance of the model highlights a nontrivial feature of the amyloid genetic landscape: The rate of nucleation of double mutants can be predicted by simple additivity of the energetic effects of single mutants (Fig. 2D).

The complete map of changes in free energy of activation for mutations in Aβ42 (Fig. 2G) reveals many interesting features of its mutational landscape. In total, 720 amino acid substitutions (86%) affect the free energy of activation of the nucleation reaction [*∆∆*G*‡* ≠ 0 kcal/mol, *Z*-test, false discovery rate (FDR) < 0.05, Benjamini-Hochberg correction] including in all positions of Aβ42. A total of 605 substitutions (72%) increase the free energy of activation (*∆∆*G*‡* > 0 kcal/mol, FDR < 0.05) to slow nucleation and 115 (14%) decrease *∆∆*G*‡* (*∆∆*G*‡ *< 0 kcal/mol, FDR < 0.05) to increase nucleation (Fig. 2G). A total of 482 substitutions (57%) cause a larger than 1 kcal/mol change in *∆*G*‡*, with 443 increasing and 39 decreasing *∆*G*‡* by >1 kcal/mol. The energy changes are very well correlated with the relative growth rates of individual substitutions (ρ = 0.94, *P* < 2 × 10^−308^; Fig. 2E and fig. S3A), further highlighting the additive nature of the assay but with an expanded dynamic range for mutations that slow nucleation (fig. S3, A to D, and table S4, particularly mutations of G-37, G-38, L-34, and M-35).

### Importance of the C terminus for Aβ42 nucleation

The complete free energy of activation landscape reveals a notable asymmetry in Aβ42: Mutations toward the C terminus (residues 29 to 42) have much larger effects on the free energy of activation than mutations before residue 29 (Fig. 2, G and H, and fig. S2D). Not all residues are structurally resolved in mature Aβ42 fibrils (*12*, *39*–*44*), but this distinction between structured and unstructured positions does not explain the difference in activation energies as mutations in many residues that are structured in mature fibrils have only small effects on the free energy of activation (Fig. 2, F and G, and fig. S3E).

The primary sequence of Aβ42 contains two hydrophobic regions: residues 17 to 21 [previously referred to as aggregation prone region 1 (APR1)] and residues 29 to 42 (APR2) (*29*, *45*–*47*). Both APR1 and APR2 form hydrophobic cores in mature fibril structures (Fig. 2, F and H, and fig. S2D) and have been proposed to be important for fibril stability (*47*). In our data, however, mutations in APR2 have much stronger effects on *G*‡ than mutations in APR1 (fig. S2E), indicating that APR2 is more important for the rate-limiting step of the Aβ42 nucleation reaction.

A parsimonious hypothesis based on this comprehensive free energy of activation measurements, therefore, is that the C terminus of Aβ42—but not more N-terminal residues that are also structured in mature fibrils—constitutes the structured region of the nucleation transition state.

### Comparison of free energy of activation changes to mature fibril stability changes

**Fig. 3.**
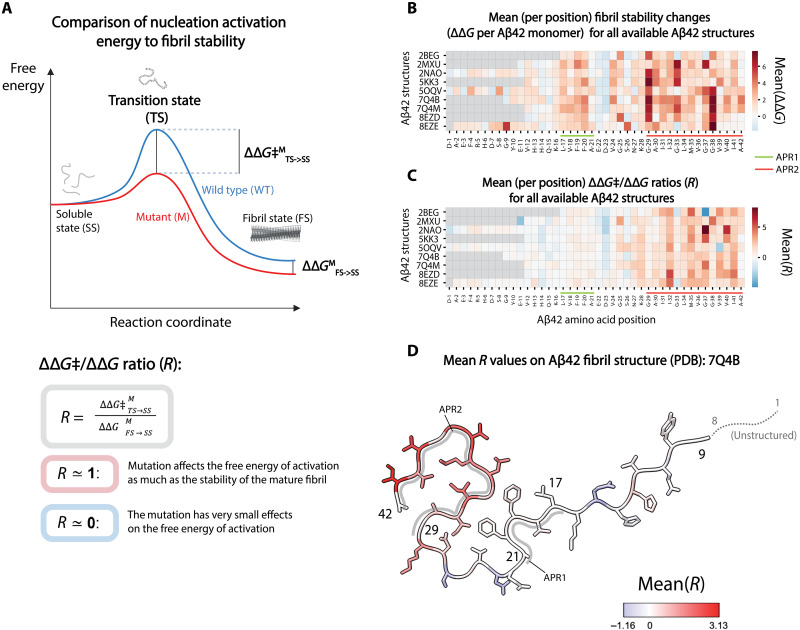
Comparing changes in free energy of activation to changes in mature fibril stability. (**A**) Free energy landscape of the amyloid nucleation reaction (top). Changes in free energy of activation (*∆∆*G*‡*^*M*^_*TS→SS*_) and in fibril stability (*∆∆*G**^*M*^_*FS→SS*_) upon mutation (M) are highlighted. Principles of *∆∆*G*‡/∆∆*G** ratio analysis (bottom). (**B**) Mean (per position) fibril stability changes (*∆∆*G** per Aβ42 monomer) as predicted by FoldX for all available Aβ42 structures for those substitutions that are later considered in ratio analysis (*∆∆*G** values are selected using the following criteria: 0.6 kcal/mol < |*∆∆G*| < 10 kcal/mol; see Methods). APR1 and APR2 are highlighted in light green and orange, respectively. (**C**) Mean (per position) *∆∆*G*‡/∆∆*G** ratios (*R*) for all available Aβ42 structures. APR1 and APR2 are highlighted in light green and orange, respectively. (**D**) Cross section of the 7Q4B PDB structure of Aβ42 fibrils with residues colored by mean *R* values per position.

We next compared the importance of residues for nucleation to their importance for the stability of mature fibrils. For each mature fibril polymorph of Aβ42, we calculated the predicted effect of every mutation on the thermodynamic stability of the fibril as the change in Gibbs free energy (*∆∆*G**) (see Methods; Fig. 3A and fig. S4) (*26*, *47*). The stability energy matrices show that both APR1 and APR2 are important for structural stability across polymorphs, with mutations in both hydrophobic cores reducing thermodynamic stability (Fig. 3B and fig. S5I).

To more formally compare the changes in free energy of activation (*∆∆*G*‡*) to changes in fibril stability (*∆∆*G**), we calculated the ratio of *∆∆*G*‡* to *∆∆*G**, an approach inspired by phi-value analysis (*22*, *48*–*51*). A *∆∆*G*‡/∆∆*G** ratio of 1 indicates that a mutation affects the free energy of activation as much as the stability of the mature fibril, whereas a ratio near zero means the mutation has very small effects on the free energy of activation. When mutations only mildly perturb a reaction path, *∆∆*G*‡/∆∆*G** ratios can be interpreted as quantifying the degree of structural conservation between the transition state and the mature fibril (Fig. 3A) (*22*, *48*–*51*).

Calculating *∆∆*G*‡/∆∆*G** ratios for all moderate effect mutations (see Methods) and all mature fibril structures of Aβ42 shows that the changes in free energy of activation are similar to changes in stability for mutations in APR2 but much smaller than changes in stability for mutations in APR1 (Fig. 3, B to D; figs. S5, A to H, and S6C; and tables S6 and S7). A simple interpretation of this result is that APR2 has a similar structure in the Aβ42 transition state as in mature fibrils, whereas APR1 does not.

The mature Aβ42 fibril structure with *∆∆*G*‡/∆∆*G** ratios closest to 1 (smallest root mean square distance to 1 across all ratios; table S8) in the APR2 region is 7Q4B, which is a cryo-EM structure of Aβ42 fibrils from familial Alzheimer’s disease brains (*12*), suggesting that the structured region of the Aβ42 transition state is most similar to this mature fibril polymorph. However, most Aβ42 mature fibril polymorphs are structured similarly in the C-terminal APR2 region (fig. S5, A to H), and the *∆∆*G*‡/∆∆*G** ratios are consistently high in this region across the polymorphs (Fig. 3C and fig. S6, A and B).

In summary, a parsimonious interpretation of the comprehensive free energy of activation measurements and comparisons to the stability of mature fibrils is that APR1 is largely unstructured in the Aβ42 nucleation transition state, whereas APR2 is structured and in a manner that is similar to the structure of this region in mature fibrils (Fig. 3, C and D).

### Massively parallel combinatorial double mutant cycles

To further probe the energy landscape of the Aβ42 nucleation reaction, we performed double mutant cycles (*20*, *21*). In double mutant cycles, the energy changes in double mutants are compared to those predicted by summing the energy changes in single mutants. For thermodynamic stability, the resulting energetic couplings can provide structural information: Whereas mutations in noncontacting positions normally result in additive changes in free energy, mutations in structurally contacting residues are often energetically coupled, causing nonadditive changes in energy when combined. Quantifying energy changes in double mutants and comparing these to energy changes in single mutants therefore provide a powerful approach to probe the structures of proteins, and this approach can also be applied to probe the structures of short-lived high energy states such as transition states (*20*, *21*, *52*, *53*).

**Fig. 4.**
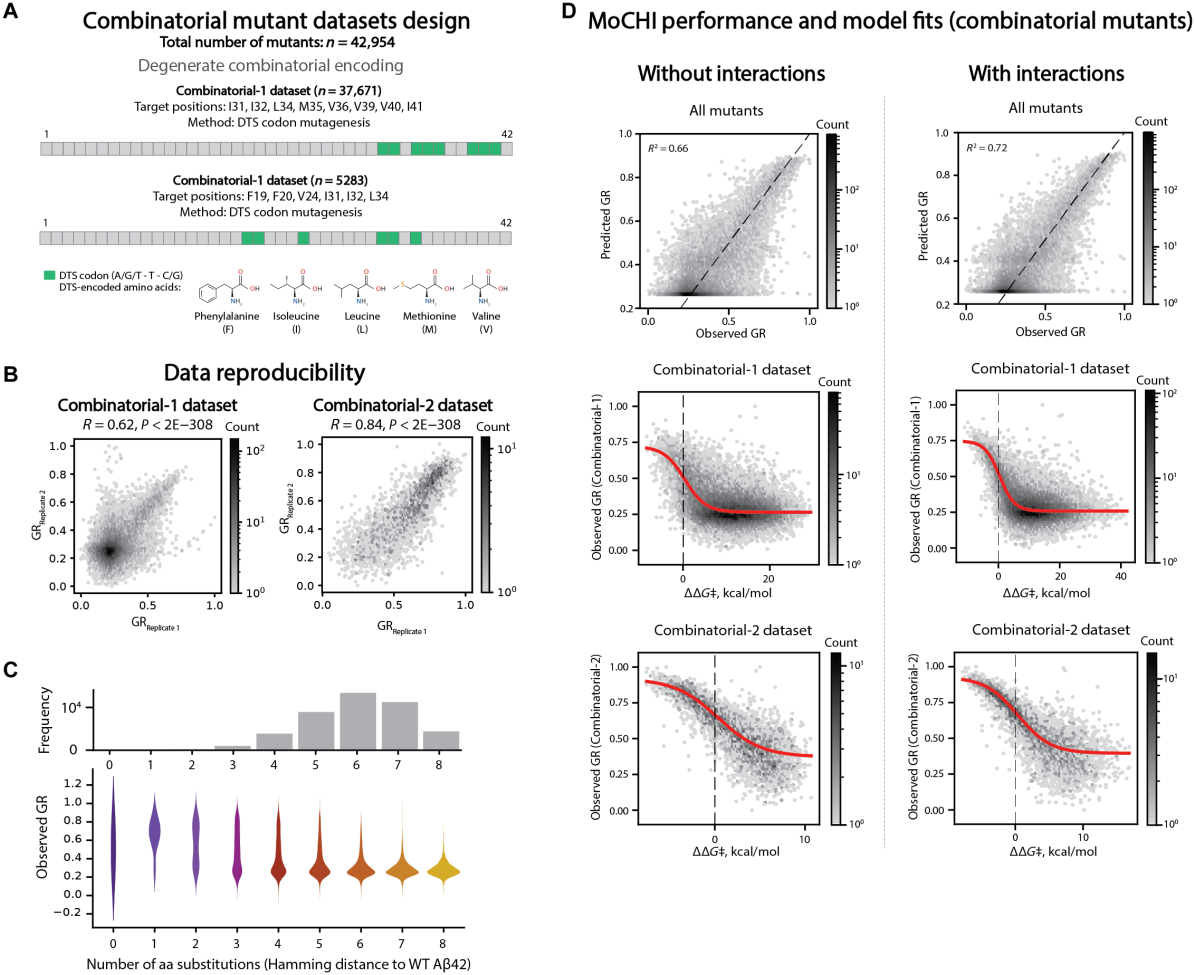
Simple genetic and energetic architecture of Aβ42. (**A**) Combinatorial Aβ42 mutant libraries’ design; DTS codons encode F (phenylalanine), M (methionine), L (leucine), I (isoleucine), and V (valine). (**B**) Inter-replicate correlations of relative growth rates (GR) for Combinatorial-1 (left) and Combinatorial-2 (right) Aβ42 combinatorial mutant libraries. Pearson’s correlation coefficients (*R*) and associated *P* values are indicated. (**C**) (Top) Histogram of the number of observed combinatorial mutants of Aβ42 at increasing Hamming distances from the WT Aβ42 (denoted by WT), for which the *x* axis is shared with the bottom panel; (bottom) Violin plot showing distributions of relative growth rates of combinatorial mutants of Aβ42 inferred from deep sequencing data versus number of amino acid (aa) substitutions. (**D**) (Left column) MoCHI performance [(top row) correlation between observed and predicted relative growth rates] for all combinatorial mutants and fits [(middle and bottom row) red trend line represents the sigmoid function fit; black vertical dashed line indicates 0 in the *x* axis] for Combinatorial-1 and Combinatorial-2 Aβ42 combinatorial mutants for model that does not account for interactions (energetic couplings). (Right column) MoCHI performance [(top row) correlation between observed and predicted relative growth rates] for all combinatorial mutants and fits [(middle and bottom row) red trend line represents the sigmoid function fit; black vertical dashed line indicates 0 in the *x* axis] for Combinatorial-1 and Combinatorial-2 Aβ42 combinatorial mutants for model that accounts for interactions (energetic couplings).

We quantified energetic couplings between mutations in Aβ42 by designing combinatorial mutagenesis libraries in which the effects of single and double mutants were quantified in many different variants of Aβ42 with different nucleation rates (Fig. 4, A, B, and D, and fig. S7A). In total, we quantified the nucleation of 42,954 combinatorial variants containing up to eight different mutations (Fig. 4C). Each specific pair of mutations is therefore tested in a median of 1024 different genotypes of Aβ42, allowing accurate measurements of both free energy changes and the energetic interactions between mutations (the energetic couplings). As for our comprehensive single mutant energy measurements, quantifying double mutants in Aβ42 variants with fast and slow nucleation rates allows more precise inference of changes in free energy of activation and measurement across an expanded dynamic range. To reduce the chances of inducing large structural changes along the reaction path, we deliberately used mutations that conserve side chain hydrophobicity (Fig. 4A and Methods). The growth rates of the combinatorial mutants were well correlated between replicate experiments (Fig. 4B). The genotype frequency landscape also matched expectations, with most genotypes in the library containing six substitutions from the wild-type (WT) Aβ42 (Fig. 4C). The median growth rates of multimutants decreased with an increasing number of mutations, but combinatorial mutants of all orders still had a wide range of rates (Fig. 4C).

### The genetic architecture of amyloid nucleation

We used MoCHI to infer the changes in free energy (*∆∆*G*‡*) in multimutants and the energetic couplings between mutations (*∆∆∆*G*‡*; Fig. 4D and fig. S7, B and C). We first considered a model in which only the effects of individual mutations are measured, and these combine additively in combinatorial mutants. Despite only containing one energy change per mutation (a total of 44 parameters), this model provides good prediction of the nucleation rates for the complete dataset (Pearson’s *R*^2^ = 0.66, evaluated by 10-fold cross-validation, total of 42,954 genotypes) (Fig. 4D, top panels). The model is a large compression of the full genotype space (~1581-fold, with 4^8^ + 4^6^ − 4^3^ possible genotypes versus 44 parameters).

We next considered a model in which pairwise energetic couplings were also inferred between all pairs of genotypes. This model provided improved predictive performance, accounting for an additional ~6% of variance (*R*^2^ = 0.72 versus *R*^2^ = 0.66 for the model without energetic couplings). The model still represents a large data compression of ~102-fold (4^8^ + 4^6^ − 4^3^ possible genotypes versus 684 model parameters).

### Sparse pairwise energetic interactions

In total, the model with pairwise energetic interactions quantifies 640 energetic couplings between 40 pairs of residues (fig. S8, A to J, and table S9). Notably, the vast majority of the energetic couplings are very small (median *∆∆∆*G*‡* = 0.3 kcal/mol), with 535/640 (84%) having an absolute value <1 kcal/mol (fig. S8K).

Less than 4% of energetic couplings have an absolute value >2 kcal/mol, with 15 positive and 9 negative couplings. This is consistent with the expectation of energetic additivity for most combinations of mutations and the small improvement in predictive performance of the model over a completely additive model (Fig. 4D).

### Energetic couplings predict a model for the Aβ42 nucleation transition state

**Fig. 5.**
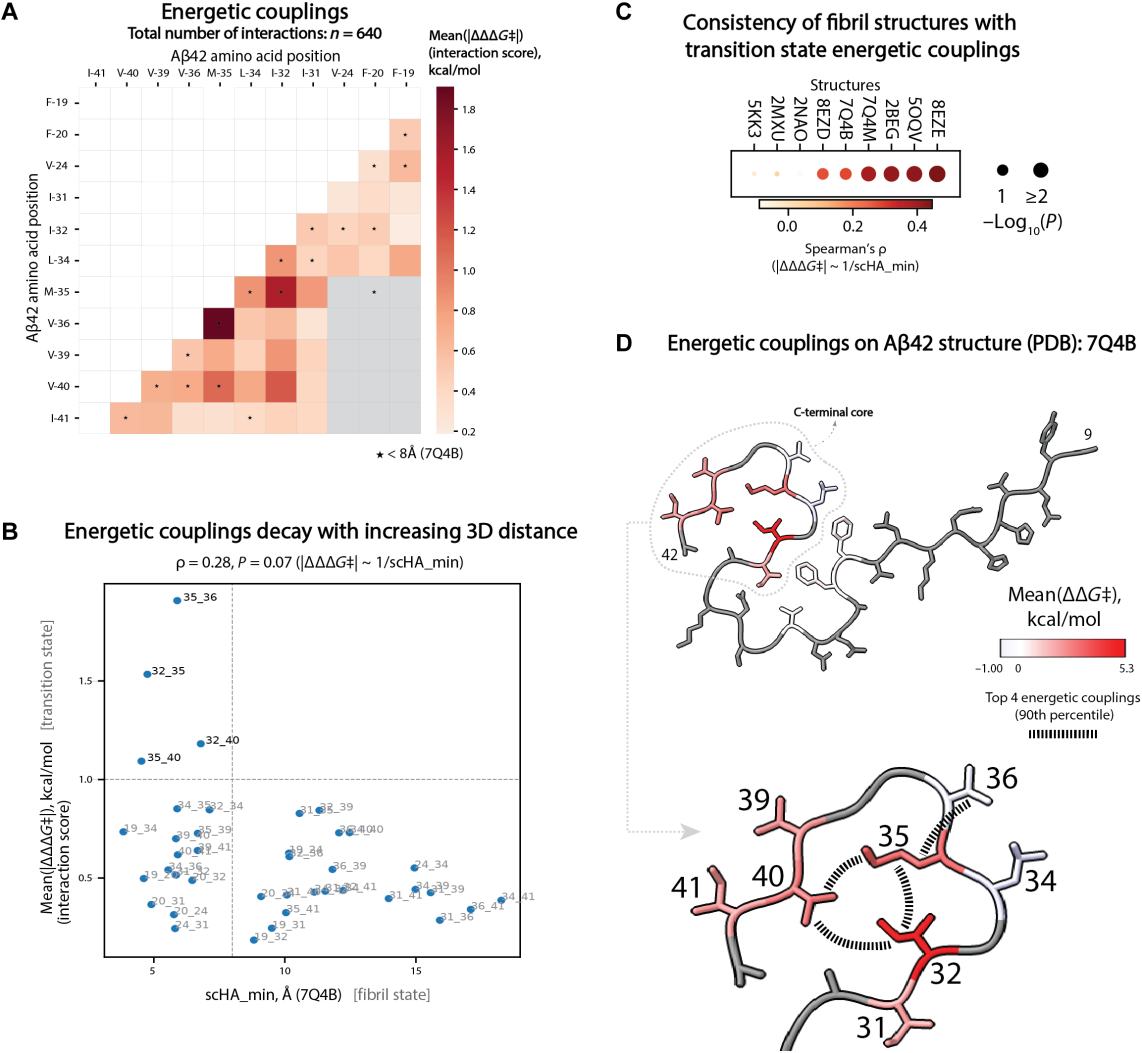
Energetic interactions in Aβ42 nucleation. (**A**) Interaction scores [mean absolute value of energetic couplings for a pair of positions, mean(|*∆∆∆*G*‡*|)] for mutagenized Aβ42 position pairs. Star symbols indicate residue pairs closer than 8 Å in the 7Q4B Aβ42 structure. (**B**) Scatterplot of interaction scores for pairs of positions and inter-residue distance for the corresponding pairs of amino acids in 3D space (scHA_min, minimum heavy atom side chain distance) as measured in the Aβ42 fibril structure 7Q4B; light gray dashed vertical line marks 8 Å; light gray dashed horizontal line marks the interaction score (mean(|*∆∆∆*G*‡*|) of 1 kcal/mol. (**C**) Dot plot representing the consistency of transition state interactions with fibril structures (2BEG, 2MXU, 2NAO, 5KK3, 5OQV, 7Q4B, 7Q4M, 8EZD, and 8EZE) according to energetic couplings and amino acid inter-residue distances; size of the dot reflects statistical significance [−log_10_(*P*)] of the correlation between interaction score [mean(|*∆∆∆*G*‡*|)] and inverse of the inter-residue distance (1/scHA_min); color of the dot represents Spearman’s ρ (correlation) coefficient. (**D**) Cross section of the 7Q4B PDB structure of Aβ42 fibrils with residues colored by the mean free energy of activation terms (*∆∆*G*‡*) inferred from the MoCHI model trained on combinatorial mutants datasets (residues with no inferred *∆∆*G*‡* are shown in gray). Positions mutagenized in combinatorial datasets (Combinatorial-1 and Combinatorial-2) are labeled on the PDB structure. Top 4 interacting position pairs (in the 90th percentile of interaction score distribution) are connected with black dashed lines.

We used the average of the absolute couplings *∆∆∆*G*‡* between different mutations in the same residues as an overall metric of the energetic nonindependence of two sites (interaction score) (*54*). The median of the interaction score distribution is 0.53 kcal/mol, with only 4/40 pairs of residues having an average coupling >1 kcal/mol (Fig. 5A and fig. S8L). All four of these strongly coupled sites are located between residues in the APR2 region: M35-V36, I32-M35, I32-V40, and M35-V40.

To compare the energetic couplings to the structures of mature fibrils, we plotted the mean absolute *∆∆∆*G*‡* values against the distance between amino acid residues in three-dimensional (3D) space (minimal side chain heavy atom distance, scHA_min) separately for each of nine Aβ42 fibril polymorphs (Fig. 5B, fig. S9A, and table S10). The couplings were correlated with the inverse of the distances in four polymorphs (8EZE, 5OQV, 2BEG, and 7Q4M, *P* < 0.02) (Fig. 5C). In Fig. 5B, we show the plot of interaction scores versus 3D structural distance for fibrils extracted from familial Alzheimer’s disease brains [Protein Data Bank (PDB) 7Q4B] (*12*), which has a notable L-shaped distribution of coupling strength versus 3D distances, with a subset of structural contacts in the mature fibrils strongly energetically coupled. Visualizing these energetic couplings on the mature fibril structure further highlights that a subset of spatially clustered structural contacts in the C-terminal APR2 region of mature fibrils is strongly energetically coupled when they are mutated (Fig. 5D and fig. S9B).

Changes in the free energy of activation of a reaction can be caused by changes in the energy of the transition state, changes in the energy of the starting state, or both (Fig. 1B). For the Aβ42 nucleation reaction, therefore, the energetic effects of mutations and the energetic interactions between them could be driven by changes in the energy of either the transition state or the starting soluble state. However, that all of the most strongly interacting pairs of positions match structural contacts in the C-terminal APR2 region of mature fibril structures suggests a parsimonious explanation of our data: Mutations and their interactions primarily affect the energy of the transition state, and this transition state is already partially structured in the C-terminal APR2 region of Aβ42. Applying Occam’s razor therefore allows us to propose a structural model for the Aβ42 nucleation transition state in which only the C-terminal APR2 region is structured and its conformation is similar to the conformation in mature amyloid fibrils.

## DISCUSSION

We have demonstrated here the feasibility of using DNA synthesis-selection-sequencing experiments to massively perturb the energy landscape of an amyloid nucleation reaction and have used the resulting data to propose a parsimonious model for the transition state of a reaction that initiates Aβ42 aggregation and so, potentially, Alzheimer’s disease.

In total, we measured the nucleation rates of >140,000 protein sequences. Using neural networks to fit energy models allowed us to quantify the change in the free energy of activation of the nucleation reaction for all 798 amino acid substitutions in Aβ42 and, in addition, the energetic interactions between 640 pairs of mutations. This is, to our knowledge, the first comprehensive measurement of changes in free energy of activation for any protein and the first large-scale measurement of energetic couplings for a kinetic process. Our work builds on pioneering studies by Fersht and others (*19*, *25*), expanding free energy of activation and energetic coupling measurements to the scale of all mutations in a sequence. The resulting energy models are simple and interpretable and represent large compressions of the genotype landscape. However, they also provide excellent predictive performance of the nucleation rates of the entire experimental dataset, revealing the reassuringly simple genetic architecture of an amyloid nucleation reaction.

We believe that the strategy taken here can be applied to massively perturb the energy landscapes of additional amyloid nucleation reactions (*31*), as well as thousands of short nucleating peptides (*30*). A key feature of our approach is the use of a kinetic selection assay where enrichments report on the rate of a reaction. This is not true for most synthesis-selection-sequencing experiments, where enrichments typically depend on thermodynamic stabilities (*35*, *55*, *56*). An important challenge for the future is the development of pooled kinetic selections for reactions other than amyloid nucleation. Approaches using microfluidics (*57*) and droplet-based selections (*58*, *59*) may facilitate this, allowing massive perturbation of the energy landscapes of additional protein reactions and high-resolution analysis of the structures of protein transition states.

Another key component of our approach is the use of double mutants and combinatorial mutagenesis. Quantifying mutational effects and interactions in variants with faster and slower kinetics expands the dynamic range of an assay and so the precision of energy measurements. The approach assumes that changes in free energy are largely additive when mutations are combined. The good predictive performance of our models even when many different mutations are combined and the consistency of nonadditive energetic couplings with structural contacts in a region of mature fibril atomic structures suggest that this is the case for Aβ42 nucleation.

Our comprehensive energy landscape identifies the residues that, when mutated, have the largest effect on the free energy of activation of the Aβ42 nucleation reaction and quantifies the energetic coupling between them. Changes in the free energy of activation of a reaction can be caused by changes in the energy of the transition state or the starting state (Fig. 1B). Formally, we cannot distinguish between these two possibilities as explanations for our data. However, we believe that the consistency between the energetic interaction map and the structure of a region of mature fibrils (Fig. 3) strongly suggests that mutations are primarily altering the energy of the transition state and that this transition state is already partially structured in the APR2 region of Aβ42 (Fig. 2).

Our study has a number of limitations. First, all our experiments use a Sup35N-Aβ42 fusion construct and we do not know the atomic structure of the Sup35N-Aβ42 fibrils that form in yeast. In future work, it will be important to determine the structure of these fibrils (including any heterogeneity) and to evaluate their similarity to Aβ42 fibrils extracted from postmortem Alzheimer’s disease samples. Second, changes in growth rate in the yeast assay may have additional causal mechanisms beyond changes in nucleation kinetics, for example, changes in Sup35N-Aβ42 expression levels. However, for all existing data, there is good agreement between the growth rates and changes in the secondary nucleation rates of Aβ42 in vitro (*28*, *29*) (Fig. 2E). Third, our analyses—and phi-value analyses more generally—assume that mutations do not cause large structural changes. To limit these structural changes, when measuring energetic couplings, we deliberately chose to use conservative mutations and to avoid mutating glycines to minimize the disruption of sharp turns in the amyloid structure. Fourth, experimental measurements of mutational effects on fibril thermodynamic stability at scale are challenging and we thus rely on FoldX calculations. However, averaging the energetic effects of 19 different mutations at each position should reduce the impact of prediction errors. Despite these four important caveats, we believe that our interpretation of the experimental data is a parsimonious working model of the energetics of an Aβ42 nucleation reaction. Both the individual mutational effects and the energetic couplings between mutations are highly consistent with the Sup35N-Aβ42 fibril transition state being structured in the C-terminal APR2 region with a conformation similar to that observed for mature fibrils extracted from postmortem Alzheimer’s disease samples.

Amyloid fibrils of Aβ42 are a defining pathological hallmark of all forms of Alzheimer’s disease, and variants in Aβ42 also cause familial Alzheimer’s disease (*3*). Moreover, the only therapeutics demonstrated to slow the progression of Alzheimer’s disease in clinical trials are antibodies targeting Aβ42 (*60*, *61*). Understanding the reaction that initiates the formation and spread of Aβ42 fibrils is a central goal of Alzheimer’s research: This reaction is the key process to stop to prevent the disease. We believe that our data and our data-driven model of the Aβ42 transition state are an important step forward in this endeavor.

## METHODS

### Library designs and construction

#### *N-terminal A*β*42 double mutant library*

To obtain the N-terminal Aβ42 double mutant library (532 possible single amino acid mutants and 151,200 possible double amino acid mutants), we used a nicking mutagenesis approach described by Wrenbeck *et al.* (*62*). The Aβ42 region with 25–base pair (bp) upstream and 21-bp downstream was amplified from the plasmid PCUP1-Sup35N-Aβ42 (*63*) [kindly provided by the Chernoff lab using oligos AB_TS_003-004 (table S11)] to introduce AvrII and HindIII restriction sites. The target region for mutagenesis was then digested and ligated (T4 DNA Ligase, Thermo Fisher Scientific) into the nicking plasmid pGJJ057, previously digested with the same restriction sites.

There are four main steps in the nicking mutagenesis protocol: (i) obtaining the single-stranded DNA (ssDNA) template, (ii) synthesis of a mutant strand by annealing and extension of mutagenic oligos (oligos AB_TS_005-032; table S11), (iii) degradation of the WT template strand, and (iv) synthesis of the second mutant strand (oligo AB_TS_033; table S11). We used 28 mutagenic oligos synthesized by IDT, each of them containing one NNK (N = A/T/C/G; K = T/G) degenerate codon at every targeted position for mutagenesis, flanked by seven upstream and seven downstream WT codons.

To obtain a double mutant library, we ran the protocol twice. In the first round, we obtained a library with 66% of single amino acid mutant and 33% of WT sequences (estimated by Sanger sequencing of individual clones). In the second round—in which we used the single mutant library as template—we obtained a library with 66% of double and 33% of single amino acid mutant sequences.

As described by Wrenbeck *et al.* (*62*), the N-terminal Aβ42 double mutant library was then transformed into 10-beta Electrocompetent *Escherichia coli* (NEB). Cells were recovered in SOC and plated on LB with ampicillin to assess transformation efficiency. A total of 2.88 million transformants were estimated, representing each variant of the library more than 18x. Fifty milliliters of overnight culture was harvested to purify the library in the nicking plasmid pGJJ057 (QIArep Miniprep Kit, Qiagen).

To clone the library back inside the PCUP1-Sup35N plasmid for selection, the library in the nicking plasmid was digested with EcorI and XbaI restriction enzymes (Thermo Fisher Scientific) for 4 hours at 37°C and purified from a 2% agarose gel (QIAquick Gel Extraction Kit, Qiagen). At this stage, the purified product was ligated into the previously digested PCUP1-Sup35N and transformed into 10-beta Electrocompetent *E. coli* (NEB). For this library, a total of 3.1 million transformants were estimated.

#### *C-terminal A*β*42 double mutant library*

To obtain a library of double mutants in the Aβ28-42 region (285 possible single amino acid mutants and 37,905 possible double amino acid mutants), we used a NNK codon mutagenesis approach. We ordered an oligo pool (IDT) containing 105 oligos of Aβ28-42 (15 amino acid positions), each with two positions containing an NNK (N = A/T/C/G; K = T/G) degenerate codon (oligo pool AB_TS_034; table S11). Each NNK degenerate codon encoded 32 possible codons, resulting in a library of 107,520 unique nucleotide sequences. Each oligo also contained 5′ (CTTTGCAGAAGATGTGGGTTCAAAC) and 3′ (TAATCTAGAGCGGCCGCCACC) constant regions for cloning purposes.

Five hundred nanograms of the oligo pool was amplified by polymerase chain reaction (PCR) (Q5 high-fidelity DNA polymerase, NEB) for 10 cycles with primers annealing to the constant regions (oligos AB_TS_037,039; table S11). The PCR product was incubated with ExoSAP-IT (Thermo Fisher Scientific) at 37°C for 1 hour and purified by column purification (MinElute PCR Purification Kit, Qiagen). This library did not contain the EcoRI restriction site between Sup35N and Aβ42, which was previously used for cloning of the shallow and N-terminal Aβ42 double mutant libraries.

In parallel, empty plasmids PCUP1-Sup35N-Aβ (1-27) (i.e., missing a specific Aβ region for mutagenesis) was constructed by PCR linearization PCUP1-Sup35N-Aβ42 using oligos AB_TS_040-041 (table S11). These was then linearized by PCR for 35 cycles (oligos AB_TS_044-045; table S11), treated with DpnI (FastDigest, Thermo Fisher Scientific) overnight, and purified from a 1% agarose gel (QIAquick Gel Extraction Kit, Qiagen).

The purified library was then ligated into 200 ng of PCUP1-Sup35N-Aβ (1-27) in a 10:1 (library:plasmid) ratio by Gibson assembly with 3 hours of incubation. The resulting product was then dialyzed for 3 hours by using a membrane filter (MF-Millipore 0.025-μm membrane, Merck) and concentrated 10X using a speed vacuum concentrator. Last, the library was transformed into 10-beta Electrocompetent *E. coli* (NEB). Cells were recovered in SOC and plated on LB with ampicillin to assess transformation efficiency. Fifty milliliters of overnight culture was harvested to purify the plasmid library (QIArep Miniprep Kit, Qiagen). A total of 1.8 million transformants were estimated, representing each variant of the library more than 47 times.

#### *Shallow A*β*42 double mutant library*

We built a library containing double mutants across the entire Aβ42 sequence (798 possible single amino acid mutants and 310,821 possible double amino acid mutants) in a previous study (*28*). Briefly, the library was obtained by error-prone PCR and cloned by digestion and ligation into the PCUP1-Sup35N plasmid (oligos AB_TS_001-002). Additional details on library construction are provided by Seuma *et al.* (*28*). For this library, a total of 4.1 million transformants were estimated, representing each variant in the library >10x.

#### *Combinatorial A*β*42 libraries*

We designed two combinatorial libraries (Combinatorial-1 and Combinatorial-2) by mutagenizing specific positions of Aβ1-42 (I31, I32, L34, M35, V36, V39, V40, and I41 in Combinatorial-1; F19, F20, V24, I31, I32, and L34 in Combinatorial-2) to a specific subset of hydrophobic amino acids. We used the DTS degenerate codon (D = A/T/G; T = C/G) to encode methionine, valine, leucine, isoleucine, and phenylalanine. For each library, we purchased 4 nmol of ultramer from IDT (oligos AB_TS_035-036; table S11) covering the target region for mutagenesis (Aβ29-42 for Combinatorial-1 and Aβ12-42 for Combinatorial-2) containing DTS codons at the abovementioned positions. The oligos also contain 5′ and 3′ constant regions for cloning purposes. The number of possible unique amino acid sequences is 390,625 for Combinatorial-1 and 15,625 for Combinatorial-2.

These libraries were amplified and cloned following the same steps as for the C-terminal Aβ42 double mutant library (oligos AB_TS_037-045). The purified Combinatorial-1 library was ligated into PCUP1-Sup35N-Aβ (1-27) whereas Combinatorial-2 into PCUP1-Sup35N-Aβ (1-11). Combinatorial-1 and Combinatorial-2 also did not contain the EcoRI restriction site between Sup35N and Aβ42, which was previously used for cloning of the shallow and N-terminal Aβ42 double mutant libraries.

A total of 0.38 million transformants were estimated for Combinatorial-2, representing each variant of the library more than 24 times. The Combinatorial-1 library was bottlenecked to 0.94 million transformants due to high complexity.

### Yeast transformation

*Saccharomyces cerevisiae* GT409 [psi-pin-] (MATa ade1-14 his3 leu2-3,112 lys2 trp1 ura3-52) strain (kindly provided by the Chernoff lab) was used in all experiments in this study. Lithium acetate transformation was used to transform each plasmid library in yeast cells, in three biological replicates. An individual colony for each transformation tube was grown overnight in 20 ml of Yeast Peptone Dextrose Adenine (YPDA) at 30°C and 200 rpm. Once at saturation, cells were diluted to OD_600_ (optical density at 600 nm) = 0.25 in 175 ml of YPDA and grown until exponential phase, for ~5 hours. Cells were then harvested, washed with Milli-Q, and resuspended in 8.5 ml of sorbitol mixture [100 mM LiOAc, 10 mM Tris (pH 8), 1 mM EDTA, and 1 M sorbitol]. Five micrograms of plasmid library, 175 μl of ssDNA (10 mg/ml, UltraPure, Thermo Fisher Scientific), and 35 ml of polyethylene glycol (PEG) mixture [100 mM LiOAc, 10 mM Tris (pH 8), 1 mM EDTA (pH 8), and 40% PEG-3350] were added to the cells and incubated for 30 min at room temperature. Heat shock was performed for 15 min at 42°C in a water bath. Cells were then harvested, washed, and resuspended in 50 ml of recovery medium (YPDA and 0.5 M sorbitol) for 1 hour at 30°C and 200 rpm. Last, cells were harvested, washed, and resuspended in 350 ml of plasmid selection medium (SC-URA 2% glucose) and grown for 48 hours. Once at saturation, cells were diluted in 200 ml of synthetic complete medium lacking uracil (SC-URA 2% glucose) to OD_600_ = 0.05 and grown until they reached the exponential phase, for ~15 hours. Last, cells were harvested and stored at −80°C in 25% glycerol. A minimum of 3.7, 3, 2.2, 0.99, and 0.43 million transformants was estimated for each of the three biological replicates of shallow Aβ42 double mutant, N-terminal Aβ42 double mutant, C-terminal Aβ42 double mutant, Combinatorial-1, and Combinatorial-2 combinatorial libraries, respectively.

### Selection experiments

Each transformation replicate was later used for selection. Tubes were thawed from −80°C, washed, and resuspended in 100 to 1000 ml of plasmid selection medium (SC-URA 2% glucose)—ensuring 100 cells each variant in the library—at OD = 0.05 and grown until exponential at 30°C and 200 rpm. Once at exponential, cells were diluted in 100 to 1000 ml of protein induction medium (SC-URA 2% glucose and 100 μM Cu_2_SO_4_) at OD = 0.05 and grown for 24 hours at 30°C and 200 rpm. After 24 hours, 25 to 50 ml of cells was harvested for input pellets and a minimum of 18.5, 75, 12, 230, and 1.5 million cells per replica (for shallow Aβ42 double mutant, N-terminal Aβ42 double mutant, C-terminal Aβ42 double mutant, Combinatorial-1, and Combinatorial-2 libraries, respectively) were plated on -URA-ADE selection medium plates (145 cm^2^, Nunc, Thermo Fisher Scientific) and incubated for 6 days (for C-terminal Aβ42 double mutant, Combinatorial-1, and Combinatorial-2 combinatorial libraries) or 7 days (shallow and N-terminal Aβ42 double mutant libraries) at 30°C. The number of cells plated ensures a minimum coverage of 10 times each variant in the library. Last, colonies were scraped off the plates and harvested for output pellets. Inputs and output pellets were stored at −20°C and later treated for DNA extraction.

### DNA extraction

Input and output pellets (three biological replicates each, six tubes in total) were resuspended in 2 ml of extraction buffer [2% Triton-X, 1% SDS, 100 mM NaCl, 10 mM Tris (pH 8), and 1 mM EDTA (pH 8)] and subjected to two cycles of freezing-thawing in an ethanol-dry ice bath and water bath at 62°C for 10 min each. A 1.5-ml solution of phenol:chloroform:isoamyl (25:24:1) was then added to the pellets and vortexed for 10 min. The aqueous phase was recovered by means of 30 min centrifugation at 3000 rpm. DNA was precipitated with 1:10 volumes of 3 M NaOAc and 2.2 volumes of 99% cold ethanol and incubated at −20°C for 2 hours. After centrifugation, pellets were dried overnight at room temperature. The next day, pellets were resuspended in TE 1X buffer [10 mM Tris (pH 8) and 1 mM EDTA (pH 8)] and treated with 10 μl of RNase A (Thermo Fisher Scientific) for 1 hour at 37°C. DNA was purified using a silica bead extraction kit (QIAEX II Gel Extraction Kit, Qiagen). Plasmid library concentration was quantified by quantitative PCR using primers annealing to the origin of the replication site of the plasmid (oligos AB_TS_046-047; table S11).

### Sequencing library preparation

Each sequencing library was prepared in a two-step PCR (Q5 high-fidelity DNA polymerase, NEB). First, the mutagenized Aβ region was amplified for 15 cycles with frameshifted primers (oligos AB_TS_048-080; table S11), using 300M (for shallow Aβ42 double mutant library), 100M (for N-terminal Aβ42 double mutant, C-terminal Aβ42 double mutant, and Combinatorial-1 combinatorial libraries), and 50M (for Combinatorial-2 combinatorial library) molecules as template for each sample (three inputs and three outputs). PCR products were treated with ExoSAP-IT (Thermo Fisher Scientific) at 37°C for 1 hour, purified by column purification (MinElute PCR Purification Kit, Qiagen), and eluted in 5 μl of TE 1X buffer (10 mM Tris and 1 mM EDTA). Four microliters of purified product was used as template for the second PCR. In this case, samples were amplified for 10 cycles with primers containing Illumina sequencing indexes (oligos AB_TS_081-111; table S11). The three input samples were pooled together equimolarly, and the same for the three output samples. The final pools were purified from a 2% agarose gel using a silica bead extraction kit (QIAEX II Gel Extraction Kit, Qiagen).

Libraries were sequenced on an Illumina HiSeq2500 sequencer using 125 paired-end reads (shallow Aβ42, N-terminal, and C-terminal Aβ42 double mutant libraries) or 150 paired-end sequencing on an Illumina NextSeq500 (Combinatorial-1 and Combinatorial-2 combinatorial libraries) at the CRG Genomics core facility.

### Data processing, relative growth rates, and error estimates

FastQ files from paired end sequencing were processed using DiMSum (*64*), an R pipeline for the analysis of deep mutational scanning data. A total of 251.6M (for shallow Aβ42 double mutant library), 352.9M (for N-terminal Aβ42 double mutant library), 106.3M (for C-terminal Aβ42 double mutant library), 109.8M (for Combinatorial-1), and 26.8M (for Combinatorial-2; 15.9M for input samples and 10.9M for output samples) paired-end reads were obtained from sequencing.

5′ and 3′ constant regions were trimmed, allowing an error rate of 20% mismatches compared to the reference sequence. Reads were aligned and sequences with a Phred base quality score below 30, and nondesigned sequences were discarded. Variants with fewer than 10 input reads in any of the replicates were discarded. Estimates from DiMSum were used to choose the filtering thresholds.

Relative growth rates and their associated error estimates (available in table S2) were also calculated using DiMSum. Relative growth rate for a specific variant i [ ${\mathrm{GR}}_{i\;\left(\mathrm{vs}\;\mathrm{WT}\right)}$ ] in the library in each biological replicate is defined in the following way:

${\mathrm{GR}}_{i\;\left(\mathrm{vs}\;\mathrm{WT}\right)}={\mathrm{ES}}_{i}-{\mathrm{ES}}_{\mathrm{WT}}\;$ , where ${\mathrm{ES}}_{i}$ is the enrichment score for the variant i , and ${\mathrm{ES}}_{\mathrm{WT}}$ is the enrichment score of the WT Aβ42, both defined as follows: ${\mathrm{ES}}_{i}=\text{log}\left(F_{i}^{\text{OUTPUT}}-F_{i}^{\text{INPUT}}\right)$.

Relative growth rates for each variant were merged using error-weighted mean of each variant across replicates and centered using the error-weighted mean frequency of WT Aβ42 synonymous substitutions. Because of a large possible sequence space (16-391K variants at the amino acid level) in combinatorial mutant datasets, WT Aβ42 was not present there, so the normalization was done using a random variant present in those datasets [namely, GGTGCAATCATCGGATTGATGGTGGGCGGTGTGGTGTTCGCG and GTTCATCATCAAAAATTGGTGTTGTTCGCAGAAGATTTGGGTTCAAACAAAGGTGCAGTC TTGGGATTGATGGTGGGCGGTGTTGTCATAGCG were used as the WT sequence parameters in DiMSum (*64*) for Combinatorial-1 and Combinatorial-2 datasets, respectively]. For visualization purposes in Figs. 2 and 4 and figs. S2, S3, and S7, relative growth rates were scaled between 0 and 1.

### Inferring changes in free energy of activation with MoCHI

To infer free energy of activation terms from relative growth rates of cells carrying nucleating Aβ42 variants, we fitted an energy model to our datasets using MoCHI (*35*, *36*). Briefly, MoCHI takes as input amino acid sequences, measured relative growth rates, and error estimates of each variant in the library. MoCHI then predicts relative growth rates for the given variants based on a particular fit (sigmoid in this case) while correcting for global nonlinearities (nonspecific epistasis) that, in this case, are due to the upper and lower limits of the growth assay. The effects of individual mutations and mutation combinations (genetic interactions) are modeled additively at the energetic level. Using the coefficients derived by the model, one then obtains the change in free energy of activation associated with each mutation for the phenotype of interest (in our case, amyloid nucleation).

For double mutant datasets (shallow Aβ42 double mutant library, N-terminal Aβ42 double mutant library, and C-terminal Aβ42 double mutant library), we used MoCHI with default parameters for a two-state model with one phenotype (nucleation) for the three double mutants datasets, using *L1* and *L2* regularization with a lambda of 10^−5^ and allowing only first-order (additive) energy terms. We evaluated the model using the held-out “fold” from the 10 times that the model was run on the dataset.

To translate (or calibrate) additive traits inferred with MoCHI (*36*) into units of energy (kcal/mol), we used experimentally measured nucleation rate constants from previously published studies (*37*, *38*) (table S1). We derived changes in free energy of activation for Aβ42 variants from the available kinetic data, using the following relationship (derived from the Transition state theory; see Fig. 1C):

${\Delta \Delta G}_{\left(i\;vs\;WT\right)}^{\text{experimental}}=RT^{*}\text{ln}\left(\;\frac{k_{WT}}{k_{i}}\;\right)$ , for Aβ42 variant i , where R is the universal gas constant and T is temperature in kelvin (303 K, as used in MoCHI model training). For the Yang *et al.* dataset (*38*), we used primary and secondary nucleation rate constants (*k_n_* and *k*_2_) reported in the study to directly calculate changes in free energy of activation for reported Aβ42 variants (D23N, E22G, E22Q, E22K, and A21G) relative to WT. For the Thacker (*37*) dataset, we derived multiplicative terms *k*_+_*k_n_* and *k*_+_*k*_2_ (primary and secondary nucleation rate constants multiplied by the rate of elongation) from the reported λ and κ values (the rate at which new fibril mass is formed via primary and secondary nucleation, respectively) and the exact model description authors provided in the supplementary material of the publication, for the reported Aβ42 variants (V18S_A21S, V40S_A42S, A-21-S, A-42-S, V-18-S, and V-40-S) relative to WT, and used these multiplicative terms in the same equation (above) to calculate the free energy of activation change. We fitted a linear regression model to be able to predict our MoCHI-inferred additive trait values from the experimentally derived ΔΔ**G*‡* values for Aβ42 variants common across the two datasets (Fig. 2E and fig. S2A), and used the resulting slope (0.233, fitting with secondary nucleation rate-derived ΔΔ**G*‡* values) to calibrate the MoCHI terms to kcal/mol units.

For combinatorial mutant datasets (Combinatorial-1 and Combinatorial-2), as WT Aβ42 variant was not present in any of the two datasets, we introduced it artificially and added to the data prior to training the MoCHI model, declaring its relative growth rate as 0 and its error estimate as an arbitrary big number, 100 in this case. We used MoCHI with default parameters for a two-state model with one phenotype (nucleation) for the two combinatorial mutants datasets, using L1 and L2 regularization with a lambda of 10^−5^, and allowing first-order (additive) and second-order (nonadditive) energy terms to account for energetic couplings. We evaluated the model using the held-out “fold” from the 10 times that the model was run on the dataset. Because we used an artificially introduced WT Aβ42 variant in MoCHI model training, we then recentered the resulting predicted free energy of activation terms. To do this, we chose all common variants between double and combinatorial mutant datasets and fit a linear regression model that inferred a slope and intercept for the free energy of activation terms in double versus combinatorial mutant datasets. We used the derived slope (0.416) and intercept (−0.155 kcal/mol) values to recenter the energy terms of the combinatorial mutants.

### Visualization of numeric values on Aβ42 structures

For purposes of visualization of numeric values (energy terms, *∆∆*G*‡/∆∆*G** ratios, and energetic couplings) on Aβ42 structures in figures, we used the following chains for each of the PDB files: B for 2BEG; D for 2MXU; C for 2NAO; D for 5KK3; F for 5OQV; E for 7Q4B; G for 7Q4M; E for 8EZD; and E for 8EZE. Molecular graphics and analyses performed with UCSF ChimeraX, developed by the Resource for Biocomputing, Visualization, and Informatics at the University of California, San Francisco, with support from the National Institutes of Health R01-GM129325 and the Office of Cyber Infrastructure and Computational Biology, National Institute of Allergy and Infectious Diseases (*65*).

### Fibril stability analyses

Inspired by phi-value analysis (*19*), we calculated free energy of activation to fibril stability energy ratios for Aβ42 by dividing our inferred free energy of activation terms *∆∆*G*‡* (Fig. 2F) by the change in free energy *∆∆*G** of fibril state structures (*47*) of Aβ42 (separately for 2BEG, 2MXU, 2NAO, 5KK3, 5OQV, 7Q4B, 7Q4M, 8EZD, and 8EZE; see table S5 for structure details). Following the example of a recent study on PI3K-SH3 amyloids, where FoldX predictions of *∆∆*G** were shown to correlate well with in vitro measurements of fibrils stability for 15 PI3K-SH3 variants (*26*), we ran FoldX on a stacked single filament tetramer fibril structures of Aβ42 peptides. For the 2NAO structure, we ran it on a stacked single filament trimer conformation as the PDB structure did not contain more than three stacked chains in any filament. We used the following Aβ42 chains: B, C, D, and E for 2BEG; A, B, and C for 2NAO; A, B, C, and D for 2MXU; A, B, C, and D for 5KK3; A, C, F, and H for 5OQV; B, D, F, and R for 7Q4B; A, C, E, and G for 7Q4M; E, F, G, and H for 8EZD; and E, F, G, and H for 8EZE. Resulting *∆∆*G** values were then divided by 4 (or 3 in the case of 2NAO) to obtain per-monomer *∆∆*G** values. In our downstream analysis, we further only considered those *∆∆*G*‡/∆∆*G** ratios, for which the predicted *∆∆*G** values were above 0.6 kcal/mol [literature-motivated threshold (*25*)] and below 10 kcal/mol, to only consider mutations that do not substantially perturb the fibril structure (Fig. 3, B and C, and figs. S4 and S5, A to H). The main assumption underlying *∆∆*G*‡/∆∆*G** ratio analysis is that the introduced mutations do not perturb these structures drastically.

### Calculation of inter-residue distances in 3D space

Distances between amino acid side chains in 3D space were calculated using the DMS2structure toolkit [available at https://github.com/lehner-lab/DMS2structure/ and published in a previous study (*52*)]. Briefly, for two given amino acids in a PDB structure, DMS2structure calculates distances between all pairs of heavy atoms (any atoms other than hydrogen) in their side chains across the two amino acids. The minimum of these distances is then reported as the minimal side chain heavy atom distance (scHA_min), further used in downstream analysis. In the absence of a side chain for glycine, the central carbon atom (C-α) is used for calculations. We specifically used contact_matrix_from_pairdistances.R script to calculate inter-residue distances for monomer conformations of Aβ42 structures and pairdistances_from_PDB_crystal.R script to calculate inter-residue distances for dimer (two monomers in different filaments facing each other) conformations of Aβ42 structures.
