# Supplementary material for: Massively parallel genetic perturbation suggests the energetic structure of an amyloid-β transition state
Source: Sci Adv. 2025 Jun 11;11(24):eadv1422. doi: 10.1126/sciadv.adv1422 (PMC12153979; doi:10.1126/sciadv.adv1422)
Supplement: Supplementary file 1 — Figs. S1 to S9 Legends for tables S1 to S11 [file sciadv.adv1422_sm.pdf]

Science Advances
AAAS

Supplementary Materials for

Massively parallel genetic perturbation suggests the energetic structure of an amyloid-β transition state

Anna Arutyunyan *et al.*

Corresponding author: Benedetta Bolognesi, bbolognesi@ibecbarcelona.eu; Ben Lehner, bl11@sanger.ac.uk

Sci. Adv. **11**, eadv1422 (2025)
DOI: 10.1126/sciadv.adv1422

The PDF file includes:

Figs. S1 to S9
Legends for tables S1 to S11

Other Supplementary Material for this manuscript includes the following:

Tables S1 to S11

Supplementary Figures

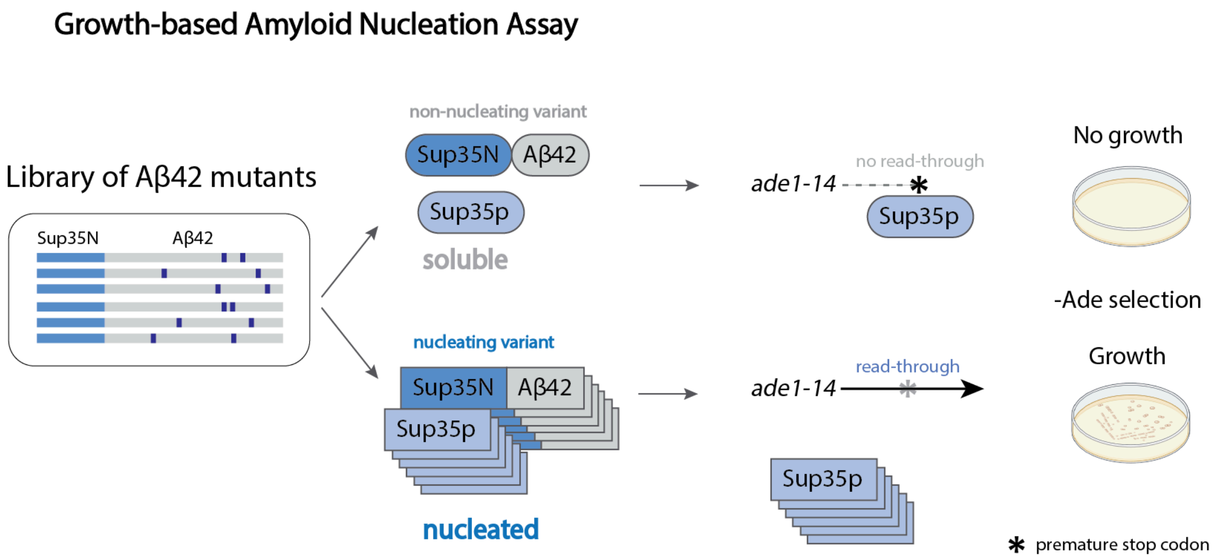

Figure S1. High throughput quantification of amyloid nucleation kinetics. Schematic overview of the amyloid nucleation assay: Aβ42, fused to the nucleation domain of Sup35 (Sup35N), seeds aggregation of the yeast prion Sup35p, causing read-through of a premature stop codon in the *ade1* reporter gene, thus allowing growth in medium lacking adenine.

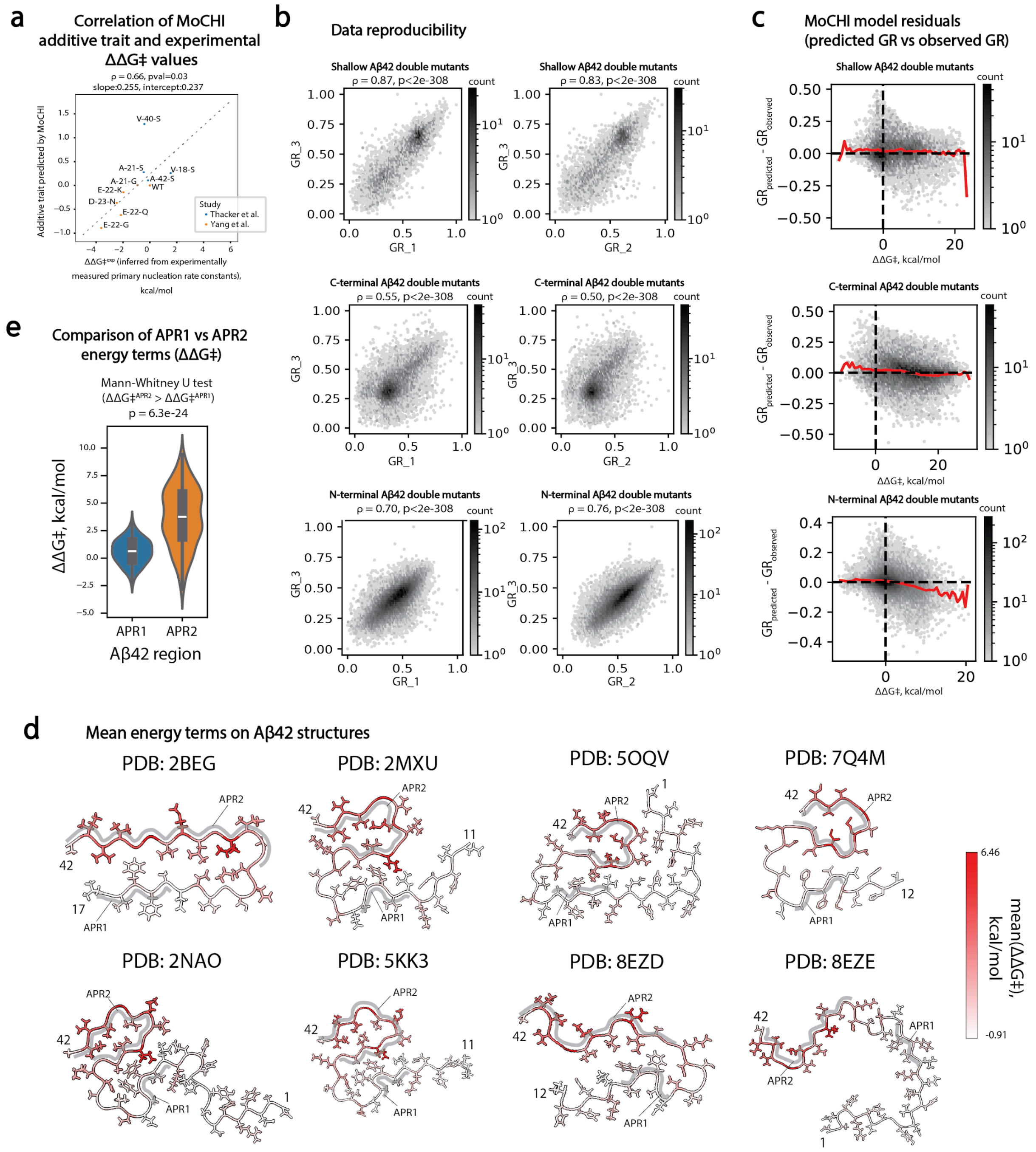

Figure S2. **Overview of double mutant libraries and inferred free energy of activation changes**. **a**, Scatterplot of additive trait values predicted by MoCHI(*34*) model trained on double mutant datasets (Y axis) and experimentally-derived ΔΔG‡ values (X axis, using primary nucleation rate constants). Dashed grey line represents linear regression fit for the data. **b**, Inter-replicate correlations of relative growth rates (GR) for shallow, C-terminal and N-terminal Aβ42 double mutant libraries (from top to bottom). Spearman's ρ (correlation) coefficients and associated p-values are reported. **c**, MoCHI residuals (predicted vs observed relative growth rates, red line is following mean residuals in 50 equally spaced bins across x axis, dashed black lines indicate 0 in both axes) for shallow, C-terminal and N-terminal Aβ42 double mutant libraries (from top to bottom). **d** Cross sections of 2BEG, 2MXU, 5OQV, 7Q4B, 2NAO, 5KK3, 8EZD and 8EZE PDB structures of Aβ42 fibrils coloured by mean ΔΔG‡ per position. Aggregation prone regions 1 (APR1) and 2 (APR2) are

highlighted in grey. **e**, Violinplot comparing inferred free energy of activation terms (ΔΔG‡) between APR1 region (AA 17-21) and APR2 region (AA 29-42) of Aβ42 ($p=6.3e-24$, one-sided Mann-Whitney U test ($\Delta\Delta G‡^{APR2} > \Delta\Delta G‡^{APR1}$)).

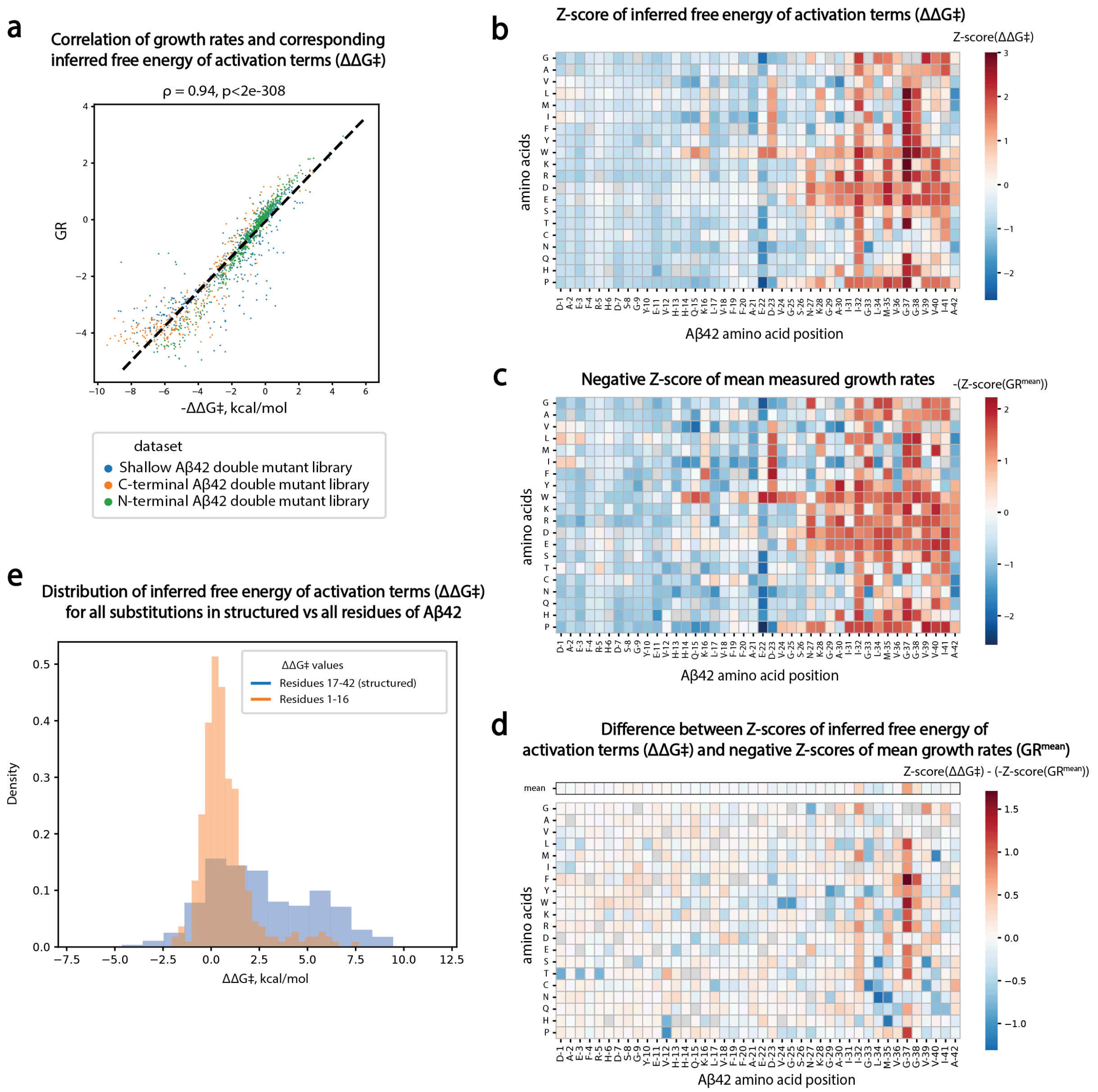

Figure S3. **Comparison of activation energies and cellular relative growth rates a**, Correlation of relative growth rates of shallow, C-terminal and N-terminal Aβ42 double mutants and corresponding inferred free energy of activation terms (ΔΔG‡). Spearman's ρ (correlation) coefficients and associated p-values are reported. Dashed black line represents linear regression fit to the data. **b**, Z-scores of inferred free energy of activation terms (ΔΔG‡) (scaled to zero mean and unit variance). **c**, Negative Z-scores of mean (across all double mutant datasets) relative growth rates (GR^{mean}) (scaled to zero mean and unit variance). **d**, Difference between Z-scores of inferred free energy of activation terms (ΔΔG‡) and negative Z-scores of mean relative growth rates (GR^{mean}). Means of difference values for each position are displayed in the top row of the heatmap (outlined in black). **e**, Distribution of inferred free energy of activation terms (ΔΔG‡) for all substitutions across only those Aβ42 positions that are structurally resolved in all available fibril structures (AA 17-42, in blue) or the rest of the positions in Aβ42 (AA 1-16, in orange).

Fibril stability changes (ΔΔG per Aβ42 monomer) predicted with FoldX (top panels) and ΔΔG‡/ΔΔG ratios (bottom panels) for all Aβ42 structures

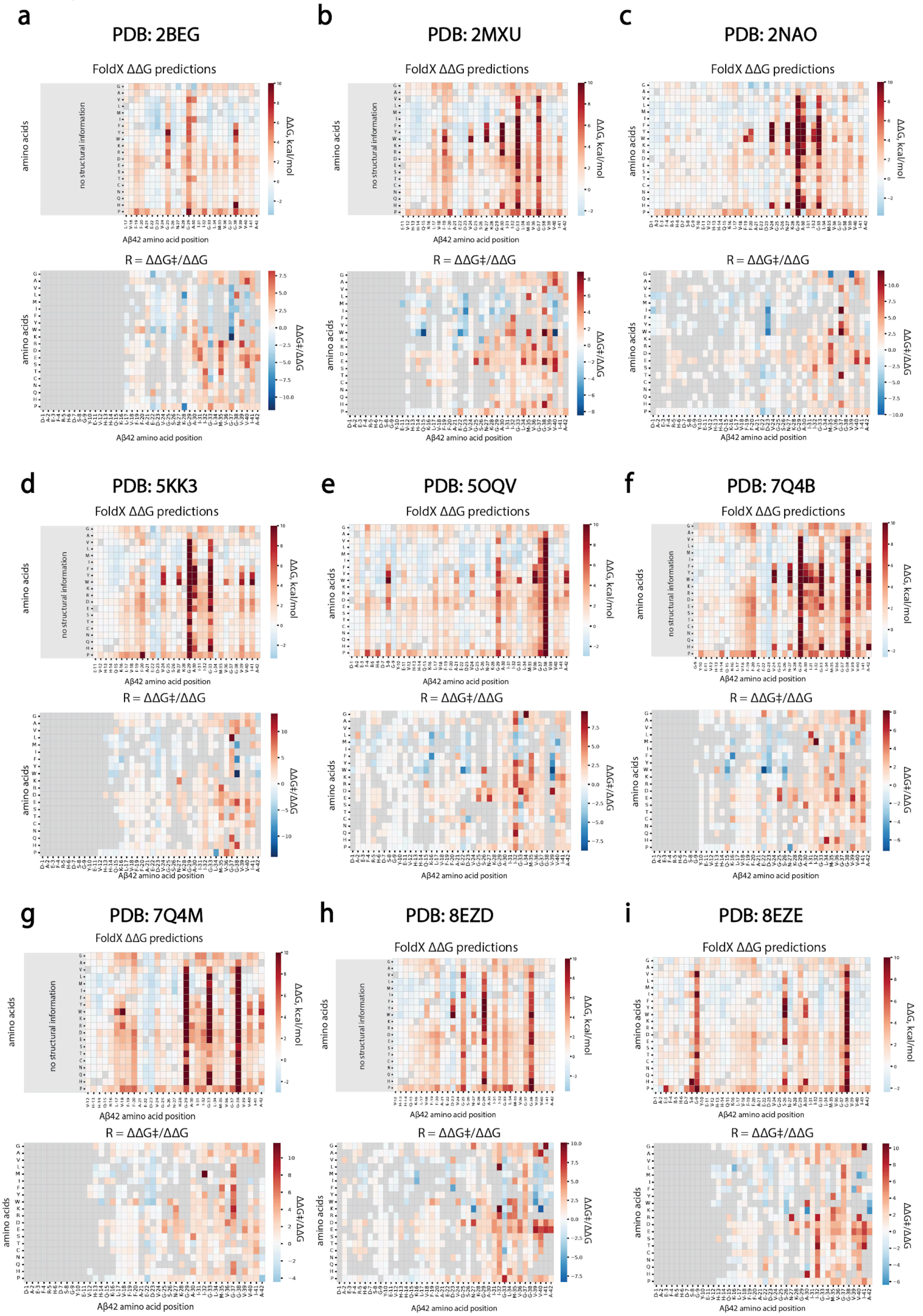

Figure S4. **Fibril stability and ∆∆G‡/∆∆G ratios (R) for all Aβ42 structures**. **a-i**, (top panels) Fibril stability changes (∆∆G per Aβ42 monomer) predicted with FoldX upon all possible substitutions and (bottom panels) R-values for structures 2BEG (a), 2MXU (b), 2NAO (c), 5KK3 (d), 5OQV (e), 7Q4B (f), 7Q4M; (g) 8EZD (h), 8EZE (i). FoldX was run using a stack of four Aβ42 monomers for all structures, apart from 2NAO where a stacked trimer was used (see Methods).

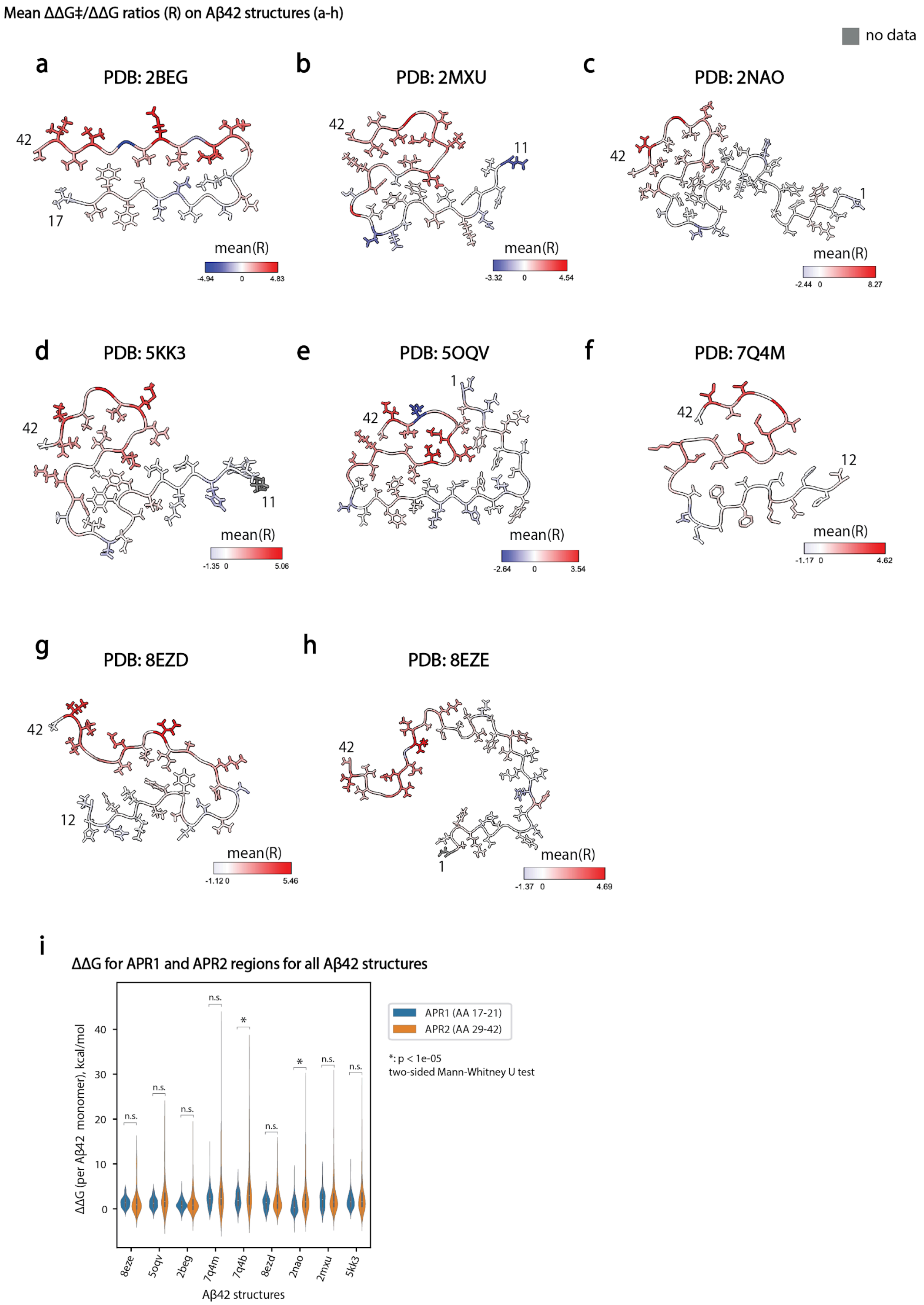

Figure S5. **Mean ΔΔG‡/ΔΔG ratios (R) on all Aβ42 structures**. **a-h**, Cross sections of PDB structures of Aβ42 fibrils coloured by mean R-values at that position: 2BEG (a), 2MXU (b), 2NAO (c), 5KK3 (d), 5OQV (e), 7Q4B (f), 8EZD (g), 8EZE (h). Residues with no R-values

are coloured in grey. **i**, Violin plot comparing fibril stability changes ($\Delta\Delta G$ per Aβ42 monomer predicted with FoldX) for all possible substitutions in APR1 vs APR2 regions of Aβ42, for all available Aβ42 fibril structures (one-sided Mann-Whitney U test statistics are reported, with significance threshold for p-value=0.001).

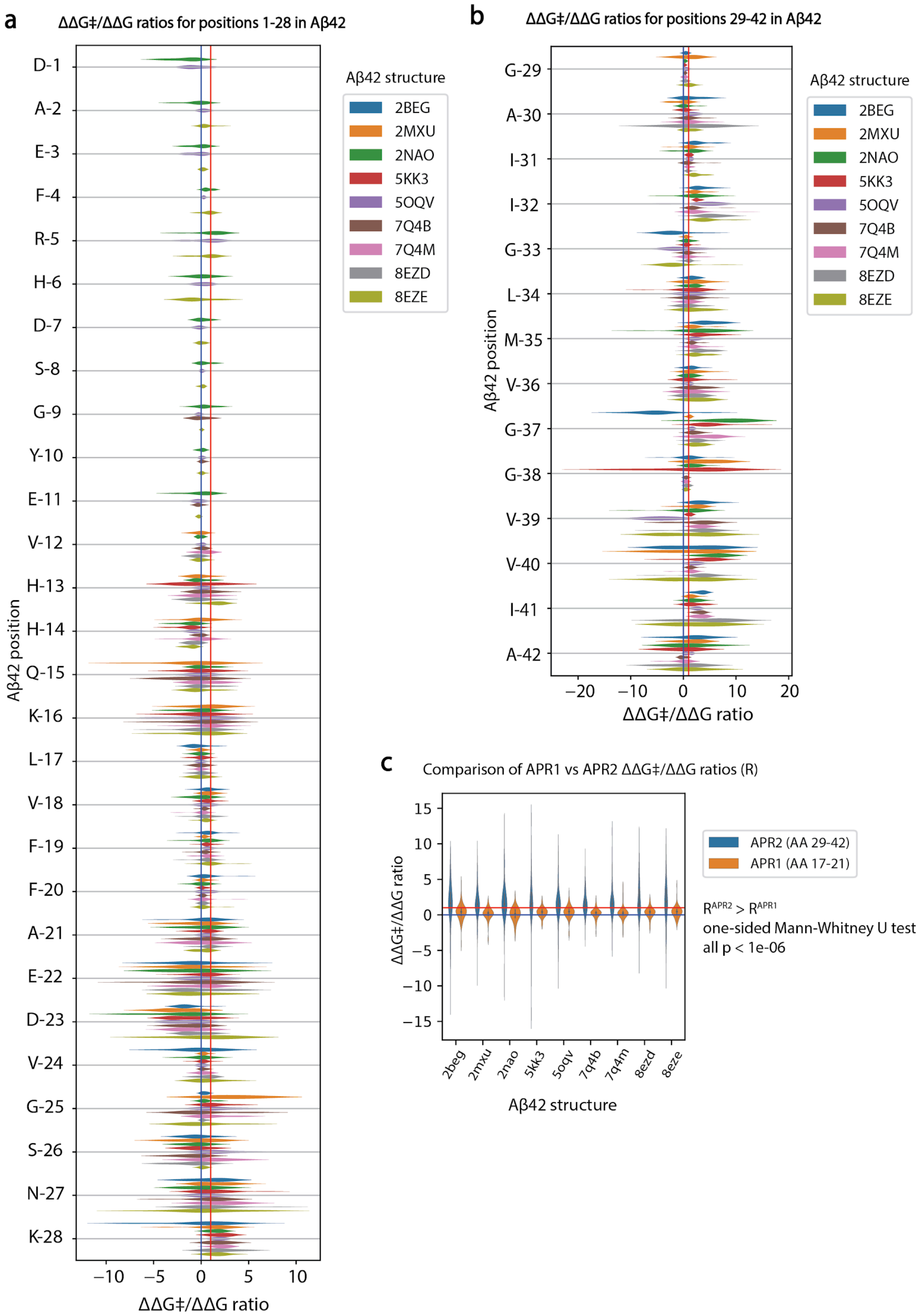

Figure S6. Overview of all R-values and comparison of APR1 and APR2 R-values. a-b, Violinplot of R-values for all the Aβ42 structures (2BEG, 2MXU, 2NAO, 5KK3, 5OQV, 7Q4B,

7Q4M, 8EZD, 8EZE) for each position at the (a) N-terminus (AA 1-27) and (b) C-terminus (AA 28-42). Red vertical lines mark R = 1, and blue vertical lines mark R = 0. **c**, Violinplot comparing APR1 region (AA 17-21) and APR2 region (AA 29-42) R-values for all the Aβ42 structures (2BEG, 2MXU, 2NAO, 5KK3, 5OQV, 7Q4B, 7Q4M, 8EZD, 8EZE). One-sided Mann-Whitney U test statistics are reported ($p<1e-06$ for all comparisons).

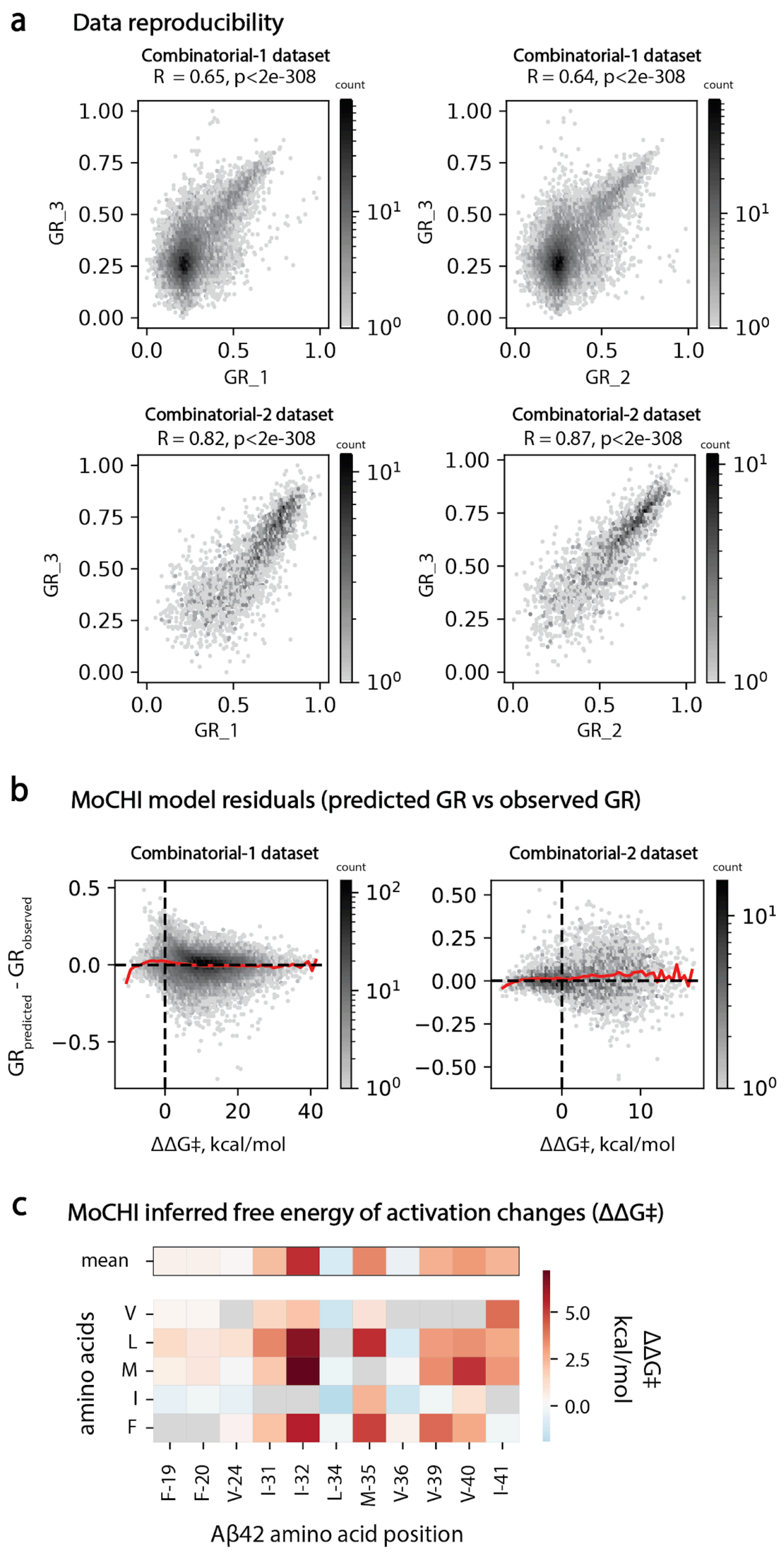

Figure S7. **Overview of combinatorial mutant libraries and energetic couplings**. **a**, Inter-replicate correlations of relative growth rates for Combinatorial-1 (top panels) and Combinatorial-2 (bottom panels) combinatorial mutant libraries. Pearson's correlation coefficients (R) and associated p-values are indicated. **b**, MoCHI residuals (predicted vs observed relative growth rates, red line is following mean residuals in 50 equally spaced bins

across x axis, dashed black lines indicate 0 in both axes) for Combinatorial-1 (left) and Combinatorial-2 (right) combinatorial mutant libraries. **c**, Heatmap displaying the inferred free energy of activation changes (ΔΔG‡) for all Aβ42 substitutions present in combinatorial mutagenesis datasets (Combinatorial-1, Combinatorial-2). Mean ΔΔG‡ values for each position are displayed in the top row of the heatmap (outlined in black).

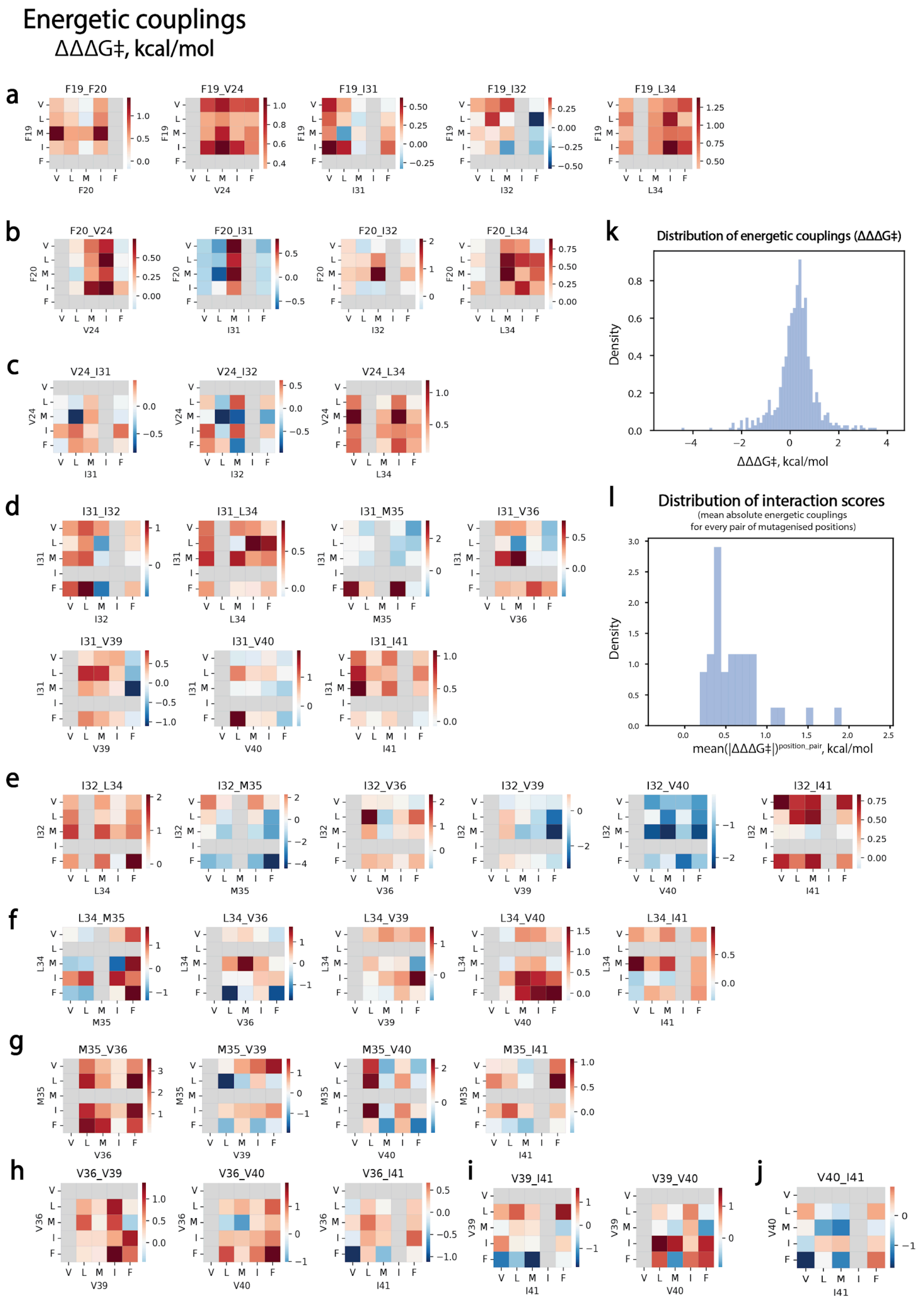

Figure S8. **Energetic coupling landscape of Aβ42**. **a-j**, Heatmaps displaying the energetic couplings between all individual mutations introduced in the combinatorial mutagenesis datasets (Combinatorial-1, Combinatorial-2) for each pair of mutated positions (F-19 (a),

F-20 (b), V-24 (c), I-31(d), I-32 (e), L-34 (f), M-35 (g), V-36 (h), V-39 (i), V-40 (j)). **k**, Distribution of 640 energetic couplings (ΔΔΔG‡) between mutations introduced in combinational Aβ42 mutant datasets. **l**, Distribution of 40 interaction scores (mean |ΔΔΔG‡|) for pairs of positions mutagenised in combinatorial Aβ42 mutant datasets.

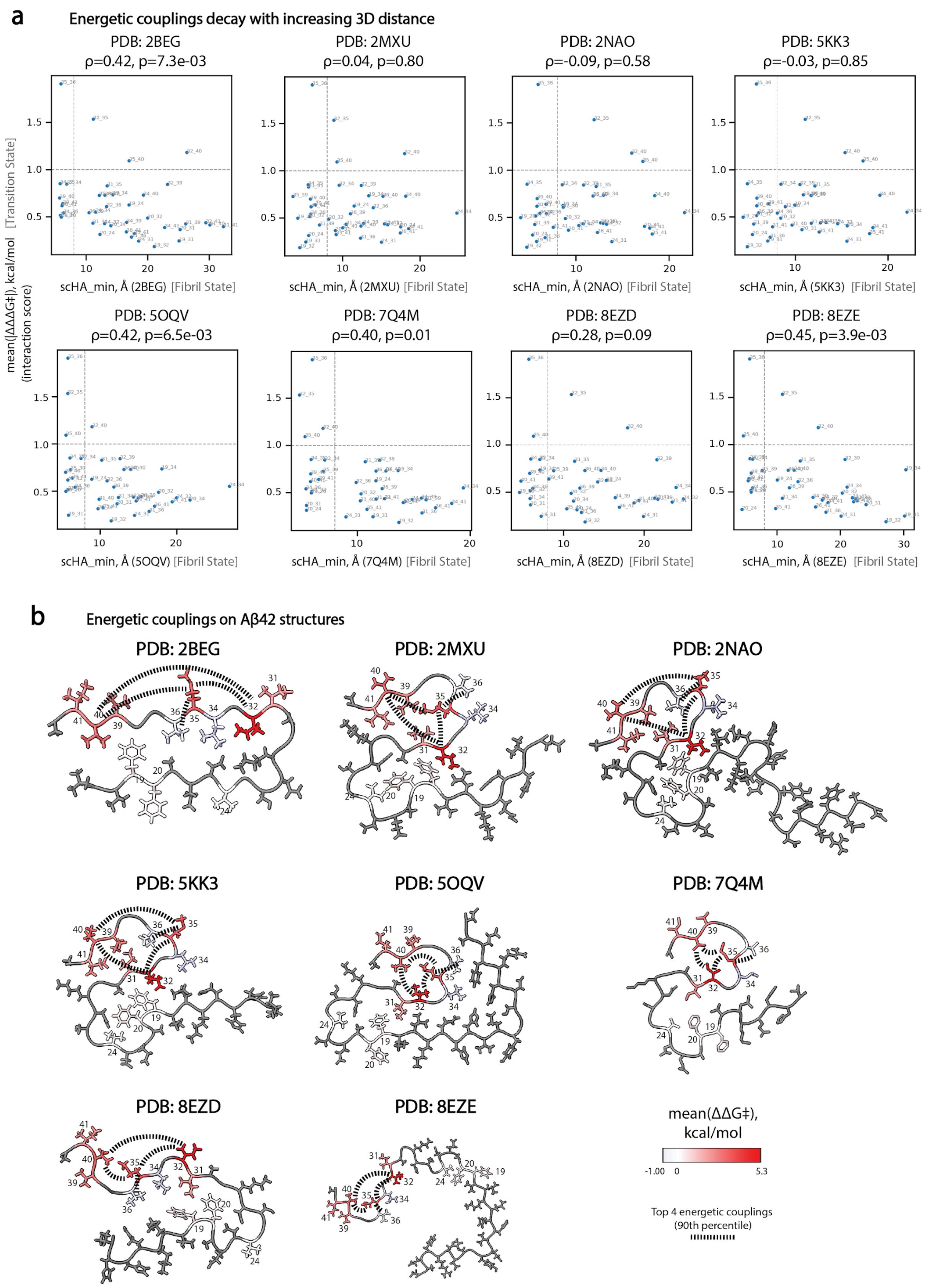

Figure S9. **Energetic couplings compared to Aβ42 fibril polymorphs**. **a**, Scatterplots of interaction scores for pairs of positions and the inter-residue distance for corresponding pairs of amino acids in 3D space (scHA_min, minimum heavy atom side chain distance) of Aβ42

structures 2BEG, 2MXU, 2NAO, 5KK3, 5OQV, 7Q4M, 8EZD, 8EZE; dashed light grey vertical line marks 8 Å, dashed light grey horizontal line marks interaction score (mean(|ΔΔΔG‡|) of 1 kcal/mol. **b**, Cross sections of PDB structures (2BEG, 2MXU, 2NAO, 5KK3, 5OQV, 7Q4M, 8EZD, 8EZE) of Aβ42 fibrils coloured by mean inferred free energy of activation terms (ΔΔG‡) from the MoCHI model trained on combinatorial mutants datasets (residues with no inferred ΔΔG‡ values are in grey). Positions mutagenised in combinatorial datasets (Combinatorial-1 and Combinatorial-2) are labelled on the PDB structure. Top 4 interacting position pairs (in 90th percentile by their interaction scores) are connected with dashed black lines.

Description of Supplementary Tables

Supplementary Table 1
Description: Primary and secondary nucleation rate constants measured independently in previous studies(*35*, *36*) for Aβ42 mutants (with each of the studies' data in separate sheets), and their corresponding relative growth rates measured in this study in the double mutants datasets (GR_shallow_double_mutants, GR_C_terminal_Ab42_double_mutants and GR_N_terminal_Ab42_double_mutants) and change in additive trait as inferred by MoCHI(*33*, *34*) (ddG‡_joint_model) trained on all double mutants datasets. For the Yang et al. dataset(*36*) primary and secondary nucleation rate constants (k_n and k_2) reported in the study are in 'kn_yang' and 'k2_yang' columns, respectively. For the Thacker(*35*) dataset, multiplicative terms k_+k_n and k_+k_2 (primary and secondary nucleation rate constants multiplied by the rate of elongation) were derived from the reported λ and κ values (the rate at which new fibril mass is formed via primary and secondary nucleation, respectively, in columns 'lambda' and 'kappa') using the exact model description authors provided in the supplementary material of the publications, and are in 'k+kn_thacker' and 'k+k2_thacker' columns, respectively.

Supplementary Table 2
Description: Relative growth rates (relative to WT) and their associated error estimates from DiMSum(*64*) for all the Aβ42 mutants (double and combinatorial) generated and analysed in this study.

Supplementary Table 3
Description: Inferred changes in free energy of activation (column 'ddG‡_scaled', in kcal/mol units) for all the substitutions in Aβ42 (column 'id') with associated outputs from MoCHI(*33*, *34*) model (fit on all the Aβ42 double mutant datasets jointly). Columns 'zscore_unaffected', 'p.adjust_mode', 'category_affected' and 'category_incr_decr_nucleation' present the statistics from the Z-test asking whether inferred ΔΔG‡ values are different from ΔΔG‡ = 0 kcal/mol (unaffected nucleation) and in which direction (> 0 or < 0). For the first entry (WT) ΔG‡ is reported.

Supplementary Table 4
Description: (ddG‡_and_GR_values sheet) inferred changes in free energy of activation (column 'ddG‡'), corresponding mean relative growth rate for all Aβ42 double mutants (for each mutant, the mean relative growth rate is calculated across all three or less Aβ42 double mutants datasets where this mutant is present, with column 'dataset' indicating which dataset the mutant is present in). (ddG‡_and_GR_Zscores sheet) Z-scores of changes in free energy of activation (X column) and Z-scores of relative growth rates necessary to reproduce heatmaps in ED Fig.3b-d (columns 'ddG‡_norm', 'neg_GR_mean_norm' and 'ddG‡_norm_minus_neg_GR_mean_norm', respectively, for panels b,c and d).

Supplementary Table 5
Description: Aβ42 structures used in analyses of the study, indicating their PDB ID, technique employed for structural determination and peptide origin. H-D - Hydrogen Deuterium, NMR - Nuclear Magnetic Resonance, SS - solid state, EM - electron microscopy, Cryo EM - cryogenic electron microscopy, MAS NMR - magic-angle spinning NMR.

Supplementary Table 6
Description: Processed FoldX output table containing predicted changes in free energy ($\Delta\Delta G$) of Aβ42 fibril structures (each sheet in the table corresponds to a given Aβ42 PDB structure). Column 'total energy' contains total $\Delta\Delta G$ predicted for a tetramer (or in case of 2NAO structure - trimer, see Methods for details) of Aβ42 fibrils, whereas column 'ddG_per_monomer' contains $\Delta\Delta G$ values predicted by FoldX for a single monomer fibril of Aβ42. All energies are in kcal/mol units.

Supplementary Table 7
Description: Ratios of free energy of activation change $\Delta\Delta G‡$ to fibril stability change $\Delta\Delta G$ (column 'ddG‡_to_ddG_ratio') for all the relevant substitutions in Aβ42 (those with $\Delta\Delta G$ satisfying the following condition: 0.6 kcal/mol < $|\Delta\Delta G|$ < 10 kcal/mol, see Methods for more details); corresponding changes in free energy of activation (column 'ddG‡') and free energy of fibril state (column 'ddG') that were used to calculate the phi values, for all available Aβ42 structures (separately in each sheet of the table). All energies are in kcal/mol units.

Supplementary Table 8
Description: Root mean square distance to 1 across all $\Delta\Delta G‡$ /$\Delta\Delta G$ ratios (R) in APR2 region of Aβ42 (AA 29-42) ('rms_indiv_ddG‡_to_ddG_ratios' column) for each PDB structure of Aβ42 used in the analysis.

Supplementary Table 9
Description: Inferred changes in free energy of activation ('ddG‡' sheet, column 'ddG‡_scaled', in kcal/mol units) and energetic couplings ('dddG‡' sheet, column 'dddG‡_scaled', in kcal/mol units) with associated outputs from MoCHI(*33*, *34*) model (fit on all the Aβ42 combinatorial mutant datasets jointly) for all the mutations and their pairwise combinations introduced in the combinatorial Aβ42 mutant datasets.

Supplementary Table 10
Description: Interaction scores (calculated as mean of absolute energetic couplings, 'mean_abs_dddG‡' column) and the corresponding 3D distance between residues ('scHA_min' column) for every pair of positions ('Ab42_position_pair' column) mutagenised in Aβ42 combinatorial mutant datasets (these data are used to produce scatterplots in Fig. 4e and ED Fig. 9a).

Supplementary Table 11
Description: Complete list of oligonucleotides employed for cloning, mutagenesis and sequencing library preparation in this study.
